# Protocol for CRISPR-based manipulation and visualization of endogenous α-synuclein in cultured mouse hippocampal neurons

**DOI:** 10.1016/j.xpro.2025.103945

**Published:** 2025-07-21

**Authors:** Leonardo A. Parra-Rivas, Rohan Sharma, Trinity E. Rust, Hannah O. Bazick, Jared Carlson-Stevermer, Mark J. Zylka, Yuki Ogawa, Subhojit Roy

**Affiliations:** 1 Department of Pathology, University of California, San Diego, 9500 Gilman Drive, La Jolla, CA, USA; 2 Aligning Science Across Parkinson’s (ASAP) Collaborative Research Network, Chevy Chase, MD 20815, USA; 3 Department of Cell Biology and Physiology, The University of North Carolina at Chapel Hill, Chapel Hill, NC, USA; 4 UNC Neuroscience Center, The University of North Carolina at Chapel Hill, Chapel Hill, NC, USA; 5 Serotiny Inc., 329 Oyster Point Boulevard, 3rd Floor, South San Francisco, CA 94080, USA; 6 Carolina Institute for Developmental Disabilities, The University of North Carolina at Chapel Hill, Chapel Hill, NC, USA; 7 Department of Neuroscience, Baylor College of Medicine, Houston, TX, USA; 8 Department of Neuroscience, University of California, San Diego, 9500 Gilman Drive, La Jolla, CA, USA

**Keywords:** Cell Biology, Cell culture, Microscopy, Molecular Biology, CRISPR, Neuroscience

## Abstract

CRISPR-Cas9 technology enables acute gene knockdown and endogenous tagging to study single-synapse function. Here, we present a protocol for depleting alpha-synuclein (α-syn) or visualizing native α-syn with an endogenously inserted fluorescent tag in cultured mouse hippocampal neurons. We describe detailed steps, including CRISPR design, virus packaging/transduction (delivery), and validation of on-/off-target editing. This protocol should be useful for assigning precise function to contentious synaptic proteins and for visualizing protein trafficking without overexpression in cultured hippocampal neurons—an established model system for synaptic biology.

For complete details on the use and execution of this protocol, please refer to Parra-Rivas et al.^1^

## Highlights

• CRISPR-Cas9 knockdown of α-synuclein in hippocampal neurons via AAV or lentivirus
• Endogenous C-terminal tagging of α-synuclein in hippocampal neurons using AAVs
• Step-by-step validation for CRISPR-Cas9 knockdown and endogenous tagging

## Abstract (editor highlights)

Publisher’s note: Undertaking any experimental protocol requires adherence to local institutional guidelines for laboratory safety and ethics.

## Graphical abstract

**Figure:**
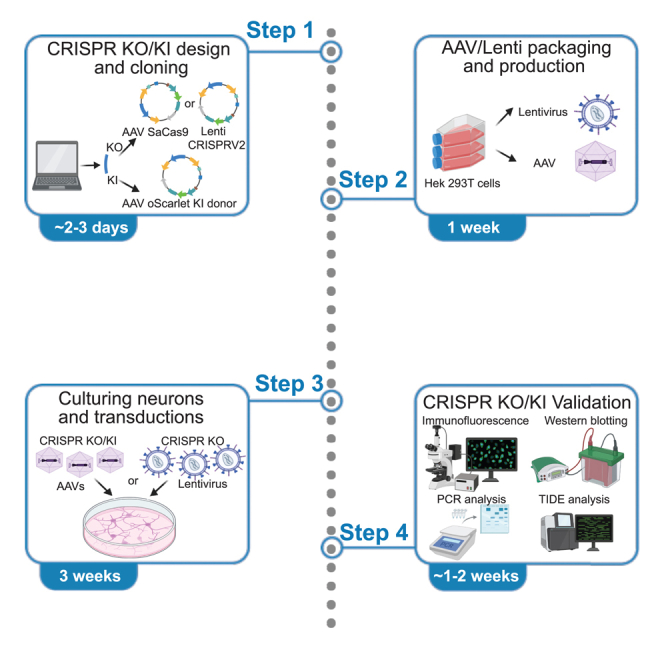

## Before you begin

The synapse is a crowded environment with thousands of proteins,^2^ and attempts to deconstruct this complex machinery by assigning functions to individual proteins has been a long-standing goal. Germline knockout (KO) studies have revealed roles for many synaptic genes,^3^^,^^4^ but compensation or unwanted phenotypes often obscure meaningful functional readouts. For instance, KO mice lacking α-syn – a cytosolic synaptic protein with disease-relevance – have relatively mild phenotypes that are likely due to compensation by β/γ synucleins.^5^^,^^6^ On the other hand, mice lacking all synucleins (α/β/γ) have unwanted changes such as early lethality, decreased synapse-size, and substantial increases in levels of other synaptic proteins^7^^,^^8^ – phenotypes that may be unrelated to α-syn knockdown. Furthermore, generating KO mice is time-consuming, and simultaneous KO of multiple genes is challenging. Some gene deletions are lethal, limiting in vivo studies. RNA-silencing approaches also face issues of incomplete knockdown, neurotoxicity, and off-target effects.^9^

Repurposing CRISPRs (Clustered Regularly Interspaced Short Palindromic Repeats) – an adaptive immune system found in bacteria and archaea – to manipulate mammalian genes has transformed our ability to evaluate function at a single-cell level.^10^^,^^11^^,^^12^ In its canonical usage, this system leverages the nuclease activity of Cas9 enzyme to generate double-strand breaks in DNA, which are subsequently repaired by an error-prone process called non-homologous end-joining (NHEJ). This repair process often introduces insertions or deletions (indels) that disrupt the coding sequence, leading to loss of protein function, typically triggering nonsense-mediated decay of the transcript.^13^^,^^14^ Alternatively, in the presence of an exogenous template, short sequences can be inserted into the host genome, in a process called homology-derived recombination (HDR).^13^^,^^14^ These approaches enable both gene knockout to evaluate function, or precise tagging for localization studies. Cultured hippocampal neurons—valued for their predictable synaptic development and similarity to in vivo synapses—have long served as a model system for studying neuronal function. Ideally, precise CRISPR-mediated manipulation in this model-system could provide insights directly relevant to the broader field of synaptic biology. However, despite the widespread use of CRISPR-Cas9 and ongoing controversies surrounding the functions of many synaptic proteins (especially cytosolic proteins^15^^,^^16^^,^^17^), few studies have applied gene editing to probe function at the level of individual synapses. This is likely due to technical challenges unique to neurons. Unlike dividing cells, where edited populations can be enriched via selection, cultured neurons are post-mitotic and difficult to transfect. As a result, achieving uniform gene editing often requires viral transduction—a method that must be carefully optimized to avoid compromising neuronal health. Moreover, HDR-based sequence insertion is inefficient in non-dividing neurons, further complicating precise genetic manipulation in these cells.

Here we describe optimized workflows for globally attenuating α-syn to undetectable levels in cultured hippocampal neurons, and also to endogenously tag α-syn in these neurons with a fluorescent marker. These knockout and knock-in (KI) tools allow investigation of native α-syn function, as well as visualization of endogenous protein trafficking, and the overall procedures should be helpful for investigating other synaptic proteins with controversial functions.

### Preparation of cell culture medium and reagents

Prepare all solutions, media, and reagents as outlined in the following section, and store them under the specified conditions until ready for use.

1. Neuronal culture media (referred to as NB/B27 medium) (store at 4°C for up to one month):
  a. Add 10 mL of B27 supplement (Gibco, Cat# 17504044) to a 500 mL bottle of Neurobasal (NB) medium (Gibco, Cat# 21103049).
  b. Add 5 mL of penicillin-streptomycin (pen-strep) (Gibco, Cat# 15140122).
  c. Add 5 mL of GlutaMAX (Thermo Fisher Scientific, Cat# 35050061).
  d. Gently invert the bottle several times to mix thoroughly.
  e. Filter-sterilize the complete medium using a 0.22 μm filter.
  f. Store the prepared medium at 4°C for up to one month.
  g. Warm to 37°C before use.

***Note:*** This formulation yields final concentrations of 1X B27, 1X pen-strep, and 1X GlutaMAX. Refer to the NB/B27 medium table in the Materials and Equipment section for additional details.

2. Neuronal lysis buffer (prepare fresh):
  a. Combine N-PER Neuronal Protein Extraction Reagent (Thermo Scientific, Cat# 87792) with 100X Protease and Phosphatase Inhibitor Cocktail (Cell Signaling, Cat# 5872) to achieve a final 1X concentration.
  b. Vortex thoroughly to ensure complete mixing and even distribution of inhibitors.
  c. Keep the buffer on ice until use.

***Note:*** Prepare this buffer fresh for each experiment to ensure maximal protein preservation.

3. Western blot running buffer (prepare fresh or up to 1–2 days in advance):
  a. Add 100 mL of 20X NuPAGE MES SDS Running Buffer (Thermo Fisher Scientific, Cat# NP0002) to a clean container.
  b. Add 1900 mL of Milli-Q H_2_O.
  c. Mix thoroughly using a magnetic stirring bar to ensure a uniform solution.
  d. Prepare fresh or up to 1–2 days in advance.
  e. Store the prepared 1X running buffer at 20°C–25°C until use.
4. Western blot transfer buffer (prepare fresh or up to 1–2 days in advance):
  a. Add 100 mL of 20X NuPAGE Transfer Buffer (Thermo Fisher Scientific, Cat# NP00061) to a clean container.
  b. Dilute with 1500 mL of Milli-Q H_2_O.
  c. Add 400 mL of methanol (Thermo Fisher Scientific, Cat# A4524).
  d. Mix extensively using a magnetic stir bar to ensure a uniform solution.
  e. Store the prepared 1X transfer buffer at 4°C until use.

***Note:*** Refer to the 1X transfer buffer table in the Materials and Equipment section for additional details.

5. PBS/Sucrose buffer (prepare fresh or up to 1–2 days in advance):
  a. Place 1.64 g of sucrose (Sigma, Cat# S7903) into a 50 mL Falcon tube.
  b. Add 36 mL of Milli-Q H_2_O.
  c. Vortex vigorously until the sucrose fully dissolves and the solution becomes homogeneous.
  d. Add 4 mL of 10X PBS (Corning, Cat# 46-013CM) to the mix and vortex.
  e. Store the buffer at RT for up to 4 weeks.

***Note:*** Refer to the PBS/Sucrose buffer table in the Materials and Equipment section for additional details.

6. Fixation buffer (prepare fresh or up to 1–2 days in advance):
  a. Place 1.64 g of sucrose (Sigma, Cat# S7903) into a 50 mL Falcon tube.
  b. Add 26 mL of Milli-Q H_2_O.
  c. Vortex thoroughly until the sucrose is completely dissolved and the solution is clear and homogeneous.
  d. Add 10 mL of 16% paraformaldehyde (PFA) stock solution (Electron Microscopy Sciences, Cat# 15710-S).
  e. Add 4 mL of 10X PBS to the mix and vortex.
  f. Store the buffer at 20°C–25°C for up to 4 weeks.

***Note:*** Refer to the fixation buffer table in the Materials and Equipment section for additional details.

7. Extraction buffer (prepare fresh or up to 1–2 days in advance):
  a. Add 4 mL of 10X PBS into a 50 mL Falcon tube.
  b. Add 35 mL of Milli-Q H_2_O.
  c. Add 1 mL of 10% Triton X-100 stock solution (Sigma, Cat# T8787) to the mixture and vortex.
  d. Store the buffer at 20°C–25°C for up to 4 weeks.

***Note:*** Refer to the extraction buffer table in the Materials and Equipment section for additional details.

8. Blocking buffer (prepare fresh or up to 1–2 days in advance):
  a. Place 0.44 g of BSA (Sigma, Cat# A8806) into a 50 mL Falcon tube.
  b. Add 34 mL of Milli-Q H_2_O.
  c. Vortex vigorously until the BSA fully dissolves and the solution becomes homogeneous.
  d. Add 4 mL of 10X PBS.
  e. Add 2 mL of FBS (Gibco, Cat# A5256701) and vortex thoroughly.
  f. Store the buffer at 20°C–25°C for up to 4 weeks.

***Note:*** Refer to the blocking buffer table in the Materials and Equipment section for additional details.

9. 1 mg/mL PEI Max stock solution (prepare in advance):
  a. Place 1 gram of PEI Max (Polysciences, Cat# 24765-1) in a glass beaker or bottle.
  b. Add 900 mL of Milli-Q or ultrapure water.
  c. Heat the mixture to 80°C for 30–60 min.
  d. Stir occasionally until the PEI fully dissolves.
  e. Allow the solution to cool to 20°C–25°C.
  f. Adjust the final volume to 1 L with Milli-Q water.
  g. Adjust the pH to 7.0 using 1N HCl or 1N NaOH.
  h. Filter the solution through a 0.22 μm sterile filter into a clean bottle.
  i. Aliquot the filtered stock into sterile 15 or 50 mL tubes and store the aliquots at −20°C for long-term use. For short-term (up to 2 months) use keep a working aliquot at 4°C.

***Note:*** The solution may initially appear cloudy but should become clear as the polymer fully dissolves. Clearly label each aliquot with the preparation date and concentration. To maintain integrity, avoid repeated freeze-thaw cycles.

10. 2 M Tris-HCl Buffer (pH 9.5) (prepare in advance):
  a. Weigh 24.2 g of Tris aminomethane (Sigma, Cat# T1503).
  b. Add the Tris to approximately 80 mL of MilliQ water in a beaker.
  c. Stir the solution until the Tris is fully dissolved.
  d. Adjust the pH to 9.5 using concentrated HCl.
  e. Bring the final volume up to 100 mL with MilliQ water.
  f. Filter-sterilize the solution using a 0.22 μm filter.
  g. Store the buffer at 20°C–25°C for short-term use or at 4°C for up to 2 weeks.

### Institutional permissions

All experimental procedures were performed in accordance with the University of California guidelines. Users of the protocol must obtain similar permissions from their respective institutions.

## Key resources table

**Table:** 

REAGENT or RESOURCE	SOURCE	IDENTIFIER
**Antibodies**		

VAMP2 mouse (1:500 dilution)	Synaptic Systems	Cat#104211BT; RRID:AB_2619758
α-syn mouse (1:500 dilution)	Synaptic Systems	Cat#610787; RRID:AB_398108
α-syn mouse (1:500 dilution)	Synaptic Systems	Cat#128211; RRID:AB_2619811
β-syn rabbit (1:500 dilution)	Abcam	Cat#ab76111; RRID:AB_1309981
SpCas9 rabbit (1:500 dilution)	Cell Signaling Technology	Cat#19526; RRID:AB_2798820
GAPDH rabbit (1:1,000 dilution)	Abcam	Cat#ab181602; RRID:AB_2630358
Synapsin-1 guinea pig (1:500 dilution)	Synaptic Systems	Cat#106104; RRID:AB_2721082
VGLUT1 rabbit (1:500 dilution)	Synaptic Systems	Cat#135303; RRID:AB_887875
Goat anti-mouse Alexa Fluor 488 (1:2,000 dilution)	Thermo Fisher Scientific	Cat#A32723; RRID:AB_2633275
Goat anti-rabbit Alexa Fluor 594 (1:2,000 dilution)	Thermo Fisher Scientific	Cat#A32740; RRID:AB_2762824
Goat anti-rabbit HRP (1:2,000 dilution)	Abcam	Cat#ab205718; RRID:AB_2819160
Goat anti-mouse HRP (1:2,000 dilution)	Abcam	Cat#ab205719; RRID:AB_2755049

**Bacterial and virus strains**		

One Shot™ Stbl3™ Chemically Competent *E. coli*	Invitrogen	Cat#C737303

**Chemicals, peptides, and recombinant proteins**		

ProFection	Promega	Cat#E1200
Lenti-X concentrator	Takara	Cat#631232
Lenti-X GoStix	Takara	Cat#631280
16% Paraformaldehyde (PFA)	Electron Microscopy Sciences	Cat#15710-S
NotI restriction enzyme	New England Biolabs	Cat#R3189L
In-Fusion Snap Assembly Master Mix	Takara	Cat#638947
DNA Ligation Kit Mighty Mix	Takara	Cat#6023
QuickExtract DNA Extraction Solution	Biosearch Technologies	Cat#QE09050
PEI Max	Polysciences	Cat#24765
Opti-MEM	Thermo Fisher Scientific	Cat#31985062
2x Ligation Mix	Takara	Cat#6023
Lucigen QuickExtract DNA extraction solution	Biosearch Technologies	Cat#QE09050
Neuronal protein extraction reagent (N-PER)	Thermo Scientific	Cat#87792
Protease/phosphatase inhibitors	Cell Signaling Technology	Cat#5872
NuPAGE LDS sample buffer	Thermo Fisher Scientific	Cat#NP007
20X NuPAGE MES SDS running buffer	Thermo Fisher Scientific	Cat#NP0002
20X NuPAGE transfer buffer	Thermo Fisher Scientific	Cat#NP00061
NuPAGE 4 to 12% Bis-Tris polyacrylamide gels	Thermo Fisher Scientific	Cat#NP0335BOX
SuperSignal West Femto Maximum Sensitivity Substrate	Thermo Fisher Scientific	Cat#FER34095X4
Restore Plus western blot stripping buffer	Thermo Fisher Scientific	Cat#46430
DC Protein Assay Kit II	Bio-Rad	Cat#5000111
TC20 automated cell counter	Bio-Rad	Cat#1450102
Tween 20	Sigma	Cat#P9416-50ML
Methanol	Thermo Fisher Scientific	Cat#A4524
0.2 μM PVDF membrane	Thermo Fisher Scientific	Cat#LC2002
Terrific Broth	Thermo Fisher Scientific	Cat#BP9728-2
Cell Strainer (70 μm)	Corning	Cat#431751
Mounting media	Electron Microscopy Sciences	Cat#17985-11
Poly-D-lysine hydrobromide (PDL)	Sigma	Cat#P6407-5MG
Boric acid	Sigma	Cat#B6768
Borax	Sigma	Cat#S9640
Sucrose	Sigma	Cat#S7903
10X PBS	Corning	Cat#46-013CM
Triton-X	Sigma	Cat#T8787
Citric acid	Sigma	Cat#C0759
Tri-sodium citrate dihydrate	Sigma	Cat#S1804
Sodium chloride	Sigma	Cat#S3014
Magnesium chloride	Sigma	Cat#68475
Tris aminomethane	Sigma	Cat#T1503
BSA	Sigma	Cat#A8806
FBS	Gibco	Cat#A5256701
Culture grade water	Millipore	Cat#4.86505
HBSS buffer	Gibco	Cat#14025092
Trypsin-EDTA	Gibco	Cat#25-200-072
B27 supplement	Gibco	Cat#17504044
Neurobasal	Gibco	Cat#21103049
Penicillin-streptomycin	Gibco	Cat#15140122
DMEM+Glutamax	Gibco	Cat#10566016

**Critical commercial assays**		

Monarch DNA gel extraction Kit	New England Biolabs	Cat#T1020S
QIAquick PCR purification Kit	QIAGEN	Cat#28104
PureLink Hi Pure Maxiprep Kit	Thermo Fisher Scientific	Cat#K210006
QIAprep Spin Miniprep Kit	QIAGEN	Cat#27104

**Deposited data**		

Vector maps and Tabular data	This paper	Zenodo: doi: https://doi.org/10.5281/zenodo.14405588

**Experimental models: Cell lines**		

Human Embryonic Kidney 293T (HEK293T)	Cellosaurus	RRID:CVCL_0063

**Experimental models: Organisms/strains**		

Neonatal (P0-P1) CD-1 IGS white (albino) mice	Charles River Laboratories	Cat#0220CD-1

**Oligonucleotides**		

α-syn forward primer (PCR#1): TGTGCTTTCTCTTCCCTCTCTG	IDT	N/A
α-syn forward primer (PCR#2): ATAACACTTCGTGCAGCACC	IDT	N/A
Reverse oScarlet primer (PCR#1 and PCR#2): ACAGGATGTCCCAGGAGAAG	IDT	N/A

**Recombinant DNA**		

AAV SaCas9	Addgene	RRID:Addgene_61591
psPAX2	Addgene	RRID:Addgene_12260
pMD2.G	Addgene	RRID:Addgene_12259
LentiCRISPR v.2	Addgene	RRID:Addgene_52961
PX552	Addgene	RRID:Addgene_ 60958
pAAV-SpCas9	Addgene	RRID:Addgene_60957
pPHP.eB	Addgene	RRID:Addgene_103005
pHelper	Agilent Technologies	Cat#240071
pMJ114	Addgene	RRID:Addgene_85995
pMJ117	Addgene	RRID:Addgene_85997
pMJ179	Addgene	RRID:Addgene_85996
α-syn:oScarlet KI donor (pLP857)	This study	RRID:Addgene_ 239403
Lenti Scramble Control (pLP16)	This study	RRID:Addgene_ 239417
Lenti α-syn (pLP17)	This study	RRID:Addgene_ 239418
AAV Scramble Control (pLP110)	This study	RRID:Addgene_ 239419
AAV α-syn (pLP111)	This study	RRID:Addgene_ 239420

**Software and algorithms**		

GraphPad Prism Software	GraphPad	RRID:SCR_002798http://www.graphpad.com/
MetaMorph Microscopy Automation and Image Analysis Software	Molecular Devices	RRID:SCR_002368https://www.moleculardevices.com/products/cellular-imaging-systems/acquisition-and-analysis-software/metamorph-microscopy#gref
CRISPick	Broad Institute	RRID:SCR_025148https://portals.broadinstitute.org/gppx/crispick/public
Nucleotide Blast	NCBI BLAST	RRID:SCR_004870https://blast.ncbi.nlm.nih.gov/Blast.cgi
BLAT tool	BLAT	RRID:SCR_011919https://genome.ucsc.edu/cgibin/hgBlat?hgsid=2143016336_Uz9QWELjorobDmSjYGqjLthfwPaU&command=start
In-Silico PCR tool	UCSC In-Silico PCR	RRID:SCR_003089https://genome.ucsc.edu/cgi-bin/hgPcr
Primer Analyzer tool	Thermo Fisher Scientific	https://www.thermofisher.com/us/en/home/brands/thermo-scientific/molecular-biology/molecular-biology-learning-center/molecular-biology-resource-library/thermo-scientific-web-tools/multiple-primer-analyzer.html
TIDE Batch tool	Bas van Steensel lab	http://shinyapps.datacurators.nl/tide-batch/
SnapGene	Dotmatics	https://www.snapgene.com/
CasOFFinder	CRISPR RGEN Tools	http://www.rgenome.net/cas-offinder/
Image Lab software version 6.1	Bio-Rad	RRID:SCR_014210

**Other**		

Mini Gel Tank system	Thermo Fisher Scientific	Cat#A25977
ChemiDoc Touch Imaging System	Bio-Rad	Cat#12003153
Inverted epifluorescence microscope (Eclipse Ti-E)	Nikon	N/A

## Materials and equipment

Prepare all solutions, media, and reagents according to the tables below, and store them under the indicated conditions until use.

### NB/B27 medium

**Table:** NB/B27 medium components

Reagent	Final concentration	Amount
B27 supplement (50X)	1X	10 mL
Penicillin-Streptomycin (10,000 μg/mL)	100 μg/mL	5 mL
GlutaMAX (100X)	1X	5 mL
Neurobasal (NB) medium	N/A	480 mL
Total	N/A	500 mL

Note: Gently invert the bottle several times to ensure thorough mixing, then filter-sterilize the complete medium. Store the prepared medium at 4°C for up to one month. Before use, warm to 37°C.

### Buffer for western blots (prepare fresh or 1–2 days in advance)

• To prepare 1X transfer buffer, refer to the table below.

**Table:** Transfer buffer (prepare fresh)

Reagent	Final concentration	Amount
NuPAGE Transfer Buffer (20X)	1X	100 mL
Methanol	N/A	400 mL
Milli-Q H_2_O	N/A	1500 mL
Total	N/A	2000 mL

Note: Mix thoroughly using a magnetic stir bar and store the buffer at 4°C until use.

### Buffers for immunocytochemistry in neurons (prepare in advance)

• To prepare 40 mL of PBS/Sucrose buffer, refer to the following table:

**Table:** PBS/Sucrose buffer

Reagent	Final concentration	Amount
Sucrose	120 mM	1.64 g
10X PBS	1X	4 mL
Milli-Q H_2_O	N/A	36 mL
Total	N/A	40 mL

Note: Vortex vigorously until the sucrose is fully dissolved and the solution becomes homogeneous. Prepare in advance and store at 20°C–25°C for up to 4 weeks.

• To prepare 40 mL of fixation buffer, refer to the following table:

**Table:** Fixation buffer

Reagent	Final concentration	Amount
Sucrose	120 mM	1.64 g
10X PBS	1X	4 mL
PFA (16%)	4%	10 mL
Milli-Q H_2_O	N/A	26 mL
Total	N/A	40 mL

Note: Vortex thoroughly to ensure complete dissolution of sucrose and obtain a clear, homogeneous solution. Prepare in advance and store at 20°C–25°C for up to 4 weeks.

• To prepare 40 mL of extraction buffer, refer to the following table:

**Table:** Extraction buffer

Reagent	Final concentration	Amount
Triton-X (10%)	0.25 %	1 mL
10X PBS	1X	4 mL
Milli-Q H_2_O	N/A	26 mL
Total	N/A	40 mL

Note: Mix well by vortexing to ensure uniformity. Prepare this solution in advance and store at 20°C–25°C for up to 4 weeks.

• To prepare 40 mL of blocking buffer, refer to the following table:

**Table:** Blocking buffer

Reagent	Final concentration	Amount
BSA	1%	0.44 g
FBS	5%	2 mL
10X PBS	1X	4 mL
Milli-Q H_2_O	N/A	34 mL
Total	N/A	40 mL

Note: Mix thoroughly by vortexing until the BSA is completely dissolved and the solution appears clear and homogeneous. Prepare in advance and store at 20°C–25°C for up to 4 weeks.

### Citrate buffer for AAV production

• To prepare 500 mL of citrate buffer, refer to the following table:

**Table:** Citrate Buffer

Reagent	Final concentration	Amount
citric acid	38.1 mM	0.44 g
sodium citrate	74.8 mM	2 mL
sodium chloride	75 mM	4 mL
magnesium chloride	100 mM	34 mL
citric acid	38 mM	3.66 g
tri-sodium citrate dihydrate	75 mM	11 g
sodium chloride	75 mM	2.2 g
magnesium chloride solution (4.9 M)	100 mM	10.2 mL
Milli-Q H_2_O	N/A	∼400 mL
**Total**	**N/A**	**500 mL**

Note: Mix all reagents thoroughly until completely dissolved. The final pH should be approximately 4.5; adjust to pH 5.0 if needed using NaOH or HCl. Filter-sterilize the buffer using a 0.22 μm filter and store at 20°C–25°C for short-term use or at 4°C for up to 2 weeks.

### Equipment

For biochemical experiments, western blots were performed using the Mini Gel Tank system (Invitrogen) and analyzed with the ChemiDoc Touch Imaging System (Bio-Rad). For immunofluorescence analysis in neurons, we use an inverted epifluorescence microscope (Eclipse Ti-E, Nikon) equipped with a CFI Plan Apochromat VC 40× oil objective (NA 1.40, Nikon) and an electron-multiplying charge-coupled device (EMCCD) camera (QuantEM:512SC, Photometrics).

## Step-by-step method details

### CRISPR KO and CRISPR KI: sgRNA design and cloning

**Timing: ∼2–3 days**

**Timing: ∼30 min (for step 1)**

**Timing: ∼30 min (for step 2)**

**Timing: ∼2 days (for step 3)**

The purpose of this step is to design and clone single guide RNAs (sgRNAs) tailored for either CRISPR-mediated gene KO or KI strategies. For CRISPR KO, sgRNAs are selected to target early coding exons of the gene of interest to induce frameshift mutations and loss of function. Depending on the experimental goals and delivery method, the sgRNAs are cloned into either AAV-based SaCas9 or lentiviral SpCas9 plasmids. For CRISPR KI, sgRNAs are carefully designed to target the last exon near the intended insertion site, enabling precise integration of exogenous sequences such as fluorescent tags while preserving endogenous gene function.

**Figure 1:**
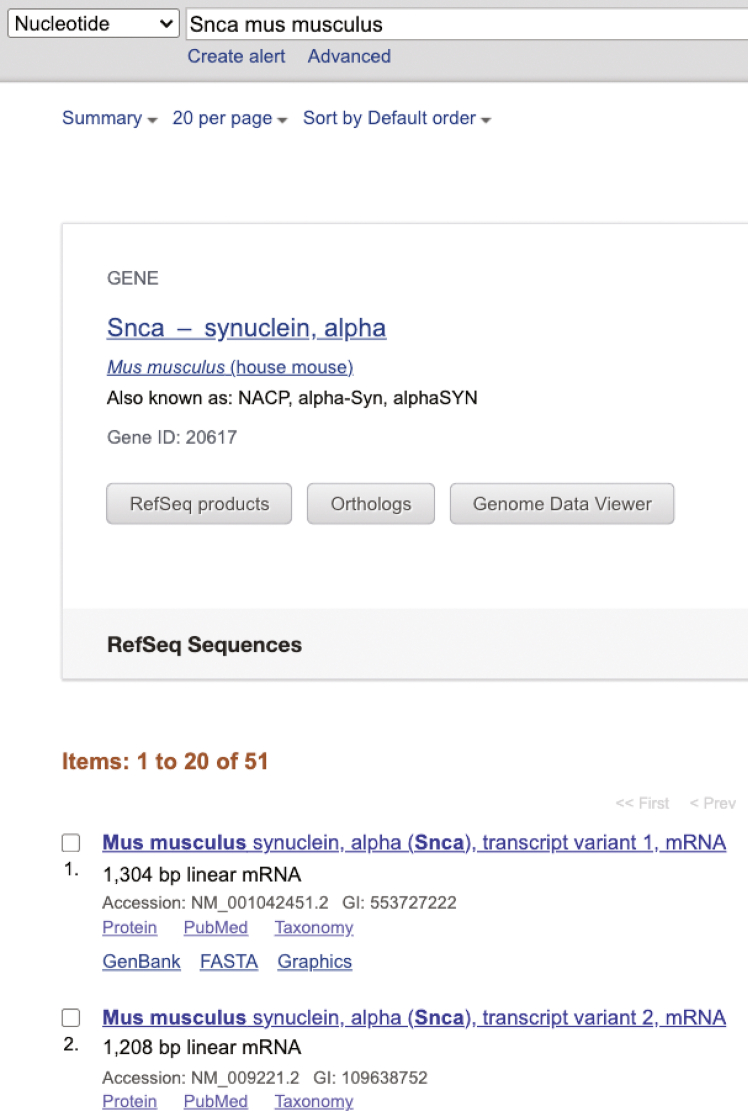
Selection of sgRNA target sites for CRISPR KI design To identify optimal sgRNA target sites, the gene of interest (e.g., *Snca*) was searched using the NCBI PubMed Nucleotide database (https://www.ncbi.nlm.nih.gov/nucleotide).
  b. Download the GenBank file for each variant, identify the last exon, and use its sequence to design the appropriate sgRNAs.
    ***Note:*** SpCas9 cleaves DNA 3 bp upstream of the PAM sequence (equivalent to 17 bp from the start of the sgRNA sequence). Thus, the cleavage site must be located within the coding region of the last exon, not beyond the stop codon or within an intron. However, the sgRNA sequence itself can extend beyond the coding region if the cleavage site remains within the coding sequence. Typically, when searching for suitable sgRNA sequences, we include up to 23 bp after the stop codon (Figure 2).

1. CRISPR KO sgRNA design.
  This section provides a step-by-step guide for designing sgRNAs for CRISPR-Cas9 genome editing. Depending on the chosen delivery method, sgRNAs can be cloned into either *Option1:* the AAV-based SaCas9 plasmid (Addgene #61591), or *Option 2:* the lentiviral LentiCRISPR v.2 SpCas9 plasmid (Addgene Cat#5261). Note that these are alternative approaches and only one delivery system is required for effective genome editing.
  a. Access CRISPick: Open CRISPick tool at (https://portals.broadinstitute.org/gppx/crispick/public)
    ***Alternative:*** Other sgRNA design tools can also be used, such as the CRISPOR web tool (https://crispor.gi.ucsc.edu/), which also provides comprehensive scoring for on-target efficiency and off-target potential.
  b. Select the reference genome.
    ***Note:*** In this example, choose *Mouse GRCm38* (Ensembl v.102).
  c. Select the appropriate CRISPR approach based on your experimental goal.
    ***Note:*** For gene knockout (CRISPRko), proceed by selecting a suitable Cas9 enzyme (SpyoCas9 or SaurCas9).
    **CRITICAL:** If you select SpyoCas9, be sure to choose the Hsu (2013) tracrRNA option during guide RNA design. This is essential for compatibility with the LentiCRISPR v.2 SpCas9 plasmid used in this protocol (Addgene #52961) and ensures optimal targeting efficiency.
    ***Note:*** The choice of Cas9 enzyme directly impacts PAM sequence compatibility and guide RNA design. PAMs are short DNA motifs required for Cas9 binding and cleavage. SpyoCas9 requires an NGG PAM sequence. In contrast, SaurCas9 recognizes NNGRR PAM sequences.
  d. Search for Target Gene: Use the “quick lookup” function in the target section to enter the gene ID. In this protocol, the target gene is *Snca*, which encodes α-synuclein.
  e. In the CRISPick Quota box, enter the number of sgRNAs you wish to design for the target gene (recommended: 2–5 sgRNAs per gene).
  f. Submit the query to generate sgRNAs.
  g. Download the results by selecting the “Picking Result File” (tab-delimited text file).
  h. Open the downloaded file in Excel. It contains summary statistics for both on-target and off-target scores, ranked by pick order.
  i. Select the top-ranked sgRNAs based on the CRISPick output, choosing at least 2–5 sgRNAs per target gene to ensure robust coverage.
    ***Note:*** CRISPick prioritizes sgRNAs using several key criteria: high on-target efficacy (predicted cutting efficiency), low off-target potential (high specificity), targeting of early exons shared across all major isoforms, proximity to the PAM sequence, avoidance of single-nucleotide polymorphisms (SNPs) and repetitive elements, and optimal GC content (40–60%).
  j. Order oligonucleotides (sgRNAs) from IDT after gRNA design.
  k. Clone the selected sgRNA sequences into the appropriate plasmid backbone using established protocols.^18^^,^^19^ Suitable options include the AAV-SaCas9 plasmid (Addgene #61591) and the LentiCRISPR v.2-SpCas9 plasmid (Addgene #52961), depending on the delivery method and experimental needs.
    ***Note:*** The guide sequences 5′-GGCAGCTGGAAAGACAAAAGA-3′ and 5′-GGTTCATGAAAGGACTTTCAA-3′ were used to target the mouse α-syn (*Snca*) gene with SaCas9 and SpCas9, respectively. The final plasmids are referred to as *AAV SaCas9 α-syn KO* and *Lenti SpCas9 α-syn KO.* For control, use a gRNA sequence that does not target any known mouse gene (5′-GACGGAGGCTAAGCGTCGCAA-3′, referred to as the “scramble control”). This same control is used for both the lentiviral SpCas9 approach and the AAV SaCas9 strategy. The guide sequences presented here were selected based on experimental testing to achieve optimal editing efficiency. We recommend designing 2–5 sgRNAs per target gene, as different editing efficiencies can be observed for each guide.
  l. Transform using One Shot Stbl3 (Invitrogen, Cat#C737303) competent cells.
  m. Pick individual colonies and inoculate into LB broth supplemented with ampicillin.
  n. Incubate 16–18 h at 37°C with shaking.
  o. Isolate plasmid DNA from 16–18 h cultures using the QIAprep Spin Miniprep Kit (QIAGEN, Cat#27104).
  p. Submit purified plasmid DNA for Sanger sequencing to confirm the presence and accuracy of the sgRNA insert.
  q. Isolate transfection-grade plasmid DNA from positive clones using the PureLink HiPure Plasmid Maxiprep Kit (Thermo Fisher Scientific, Cat#K210006) (https://assets.thermofisher.com/TFS-Assets/LSG/manuals/purelink_hipure_plasmid_dna_purification_man.pdf).
2. CRISPR KI sgRNA design.
  The following section provides step-by-step instructions for designing guide RNAs (gRNAs) to achieve homology-independent KI at the C-terminus of the gene of interest and insert an exogenous tag, such as a fluorescent marker, at this location. The same approach can also be applied to target the N-terminus. Although this protocol utilizes SpCas9, the overall steps should be similar to those of other Cas9 variants.
  a. Search for a gene of interest in Pubmed Nucleotide (e.g., *Snca*) (Figure 1).
    ***Note:*** If multiple gene variants are found, download all relevant transcript sequences and confirm the identity and structure of the last exon. For KI design, choose sgRNAs that cut as close as possible to the intended insertion site. The best target is one that combines high on-target activity, low off-target risk, and proximity to the desired KI site — preferably within an exon shared across all relevant isoforms.

**Figure 2:**
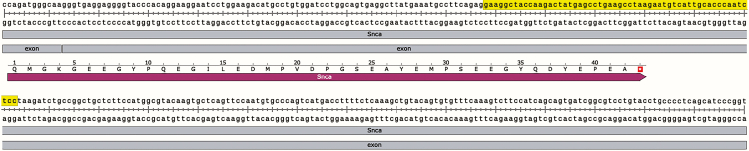
Strategy for sgRNA design targeting the last exon of a gene To design CRISPR KI sgRNAs, GenBank files for each transcript variant were downloaded and aligned to identify the final exon. A representative sequence from the last exon of *Snca* (highlighted) was used as the template for sgRNA selection.
    Example: A 56-bp sequence from the last exon of *Snca* used for sgRNA design: gaaggctaccaagactatgagcctgaagcctaagaatgtcattgcacccaatctcc.
  c. On the CRISPOR website (https://crispor.gi.ucsc.edu/), input the target sequence and select the appropriate reference genome (Figure 3).
    ***Note:*** The following reference sequences are commonly used for sgRNA design, though other versions may also be suitable:
    i. Mouse: *Mus musculus* - Mouse UCSC Jun. 2020 (GRCm39/mm39).
    ii. Rat: *Rattus norvegicus* - Rat NCBI GCF_015227675.2 (mRatBN7.2).
    iii. Human: *Homo sapiens* -Human UCSC Dec. 2013 (GRCh38/hg38).

**Figure 3:**
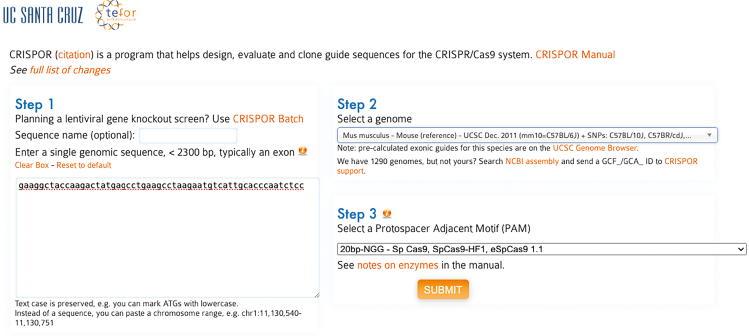
sgRNA design using CRISPOR To identify candidate sgRNAs for CRISPR KI, the target DNA sequence from the final exon was entered into the CRISPOR web tool (https://crispor.gi.ucsc.edu/), and the appropriate reference genome was selected. CRISPOR provides a ranked list of sgRNAs based on predicted efficiency and off-target scores.
  d. Review the results to identify candidate sgRNA sequences.
  e. Select sgRNA sequences for KI based on the “MIT Specificity Score” and the target region. The MIT Specificity Score ranges from 0 to 100, with higher scores indicating greater specificity (Figure 4).
    ***Note:*** In our KI vectors, the KI tag includes stop codons, and consequently the genomic sequence following the tag will not be translated. Targeting too far from the stop codon may disrupt longer sequences in the last exon, which could increase the risk of mis-localization or dysfunction of the target protein. We typically design more than two sgRNAs per target. Ideally, choose a sgRNA with a high MIT Specificity Score that targets close to the native stop codon. If this isn't feasible, design one sgRNA with a relatively high MIT Specificity Score that is targeted further away from the stop codon, along with another sgRNA that targets closer to the stop codon, even if its specificity score is lower. Avoid sequences marked as “Inefficient” or “Not with U6/U3”. For Snca, use the sequence 5′-AGGCTTCAGGCTCATAGTCT-3′, which disrupts the final six amino acids of the encoded protein.

**Figure 4:**
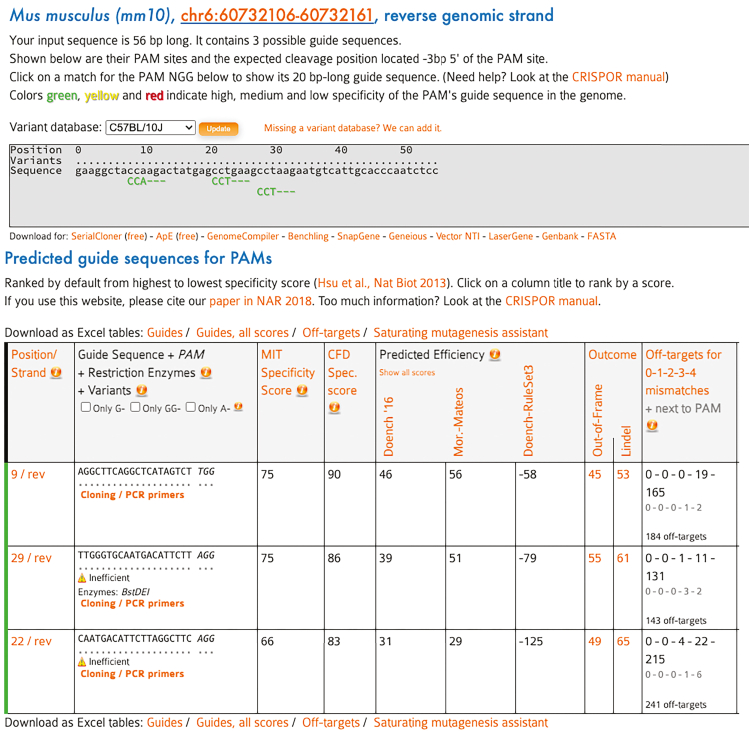
Identification of candidate sgRNA sequences Following CRISPOR analysis, sgRNA candidates were ranked based on predicted on-target efficiency, specificity scores, and potential off-target effects. Ideal sgRNAs are located as close as possible to the intended KI site, within the final exon, and exhibit high specificity with minimal off-target risk. Sequences with high MIT specificity and CFD scores were prioritized for further testing.
  f. Design a pair of complementary oligonucleotides for cloning into the CRISPR plasmid backbone.
  g. Add a 5′ overhang (cacc) to the forward oligo and a 5′ overhang (aaac) to the reverse oligo to enable directional cloning into the CRISPR plasmid backbone.
    ***Note:***:These overhangs ensure correct orientation of the sgRNA insert during ligation and are required for compatibility with the BsmBI (Esp3I) cloning sites in commonly used CRISPR vectors such as LentiCRISPR v.2 SpCas9.
    **CRITICAL:** Exclude the PAM sequence (NGG) from both oligonucleotides during design. Including the PAM sequence in the oligos can lead to unintended Cas9 cleavage of the plasmid.
  h. Order the following two oligonucleotides:
    i. Snca_KI_F: 5′-caccgAGGCTTCAGGCTCATAGTCT-3’.
    ii. Snca_KI_R: 5′-aaacAGACTATGAGCCTGAAGCCTc-3’.
3. CRISPR KI: cloning donor vector including sgRNA and fluorescent tag.
  This section outlines the CRISPR knock-in (KI) cloning strategy used to generate customized CRISPR-Cas9 vectors for targeted genome editing. Specifically, it describes the construction of a PX552-based KI plasmid designed to insert a fluorescent tag—such as oScarlet—at the *Snca* locus using the Homology-independent Universal Genome Engineering (HiUGE) approach. It also details vector modifications, including removal of the original expression cassette and insertion of custom sequences, as well as Cas9 expression via a modified pAAV-SpCas9 plasmid. Together, these steps produce tailored tools for precise and efficient genome engineering.
  a. Use a CRISPR-Cas9 knock-in (KI) vector based on the PX552 plasmid backbone (Addgene #60958) to insert a fluorescent tag at the *Snca* locus.
  b. Design the vector following the Homology-independent Universal Genome Engineering (HiUGE) strategy, incorporating study-specific modifications.^20^
  c. Choose a fluorescent tag for insertion; in this study, use the red fluorescent protein oScarlet. Alternatively, substitute a different fluorescent tag using the same vector backbone.
  d. Digest the PX552 plasmid with the NotI restriction enzyme to remove the original expression cassette flanked by ITR sequences.
  e. Insert the customized fluorescent sequence into the NotI-digested PX552 backbone, as illustrated in Figure 5 (refer to the figure legend for details on plasmid elements). The obtained construct will be referred to as AAV α-syn:oScarlet KI donor throughout the rest of the protocol.
    ***Note:*** Access the finalized AAV α-syn:oScarlet KI donor vector and empty versions (ORF-0, ORF-1, ORF-2) via Zenodo (DOI: 14405588).

**Figure 5:**
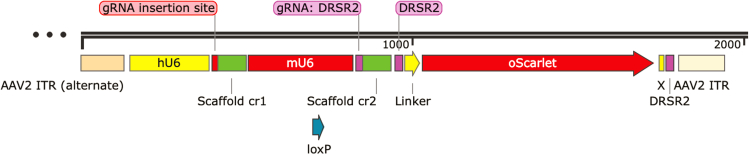
Schematic of CRISPR KI vector design and key elements hU6: Human U6 promoter, amplified from the pMJ117 plasmid (Addgene plasmid #85997; a gift from Jonathan Weissman), drives robust expression of the single guide RNA (sgRNA) in mammalian cells. gRNA Insertion Site: Site cleaved by the *BbsI* restriction enzyme to allow insertion of the sgRNA sequence of interest. Scaffold cr1: Codon-optimized sgRNA scaffold sequence, amplified from the pMJ114 plasmid (Addgene plasmid #85995; a gift from Jonathan Weissman), forms the structural component of the sgRNA and facilitates Cas9 binding. mU6: Mouse U6 promoter, amplified from the pMJ179 plasmid (Addgene plasmid #85996; a gift from Jonathan Weissman), drives expression of a second sgRNA cassette. gRNA (DRSR2): sgRNA sequence targeting the donor recognition sequence R2 (DRSR2): 5′-GCGATCGTAATCACCCGAGT-3′,used for homology-independent targeted integration.Scaffold cr2: Codon-optimized sgRNA scaffold sequence, amplified from the pMJ179 plasmid (Addgene plasmid #85996; a gift from Jonathan Weissman, used in conjunction with the DRSR2-targeting gRNA to support Cas9 function. DRSR2: Target site recognized by the gRNA: DRSR2, 5′-GCGATCGTAATCACCCGAGTGGG-3′ to enable targeted cleavage and integration of the KI cassette. Linker: Flexible amino acid linker (N-GGGGSGGGGSGGGGS-C) inserted between the targeted protein and oScarlet to minimize steric hindrance and preserve protein function. Sequence Between the Linker and oScarlet: Distinct sequences were inserted between the linker and oScarlet to preserve the correct reading frame in each construct. The sequences are as follows: ORF-0, 5′-CCTCGA-3’; ORF-1, 5′-CTCGA-3’; ORF-2, 5′-CGCTCGA-3’.oScarlet: Codon-optimized red fluorescent protein (RFP) used as a KI tag, It enables visualization of the tagged endogenous protein in live or fixed cells, amplified from the pAAV-Ef1a-oScarlet plasmid (Addgene plasmid #137135; gift from Karl Deisseroth). X: Sequence containing multiple stop codons in all three reading frames to ensure translational termination where appropriate.
  f. Express Cas9 for KI experiments using a modified pAAV-SpCas9 plasmid (Addgene #60957; provided by Feng Zhang).
    ***Note:*** The HA tag in the pAAV-SpCas9 plasmid was removed to prevent interference with downstream applications.
    **CRITICAL:** To proceed with the CRISPR KI strategy, it is crucial to select the appropriate backbone vector that aligns with the ORF between the genomic sequence and the KI tag. If the cleavage site occurs between two codons, it is classified as ORF +0, allowing the KI tag to be inserted in-frame without modification. However, if the cleavage occurs within a codon—separating the first nucleotide from the remaining two—it is classified as ORF +1. If the cleavage separates the first two nucleotides from the third, it is classified as ORF +2 (Figure 6). For ORF +1 and ORF +2, one or two nucleotides, respectively, must be added before the KI tag to maintain the correct reading frame.

**Figure 6:**
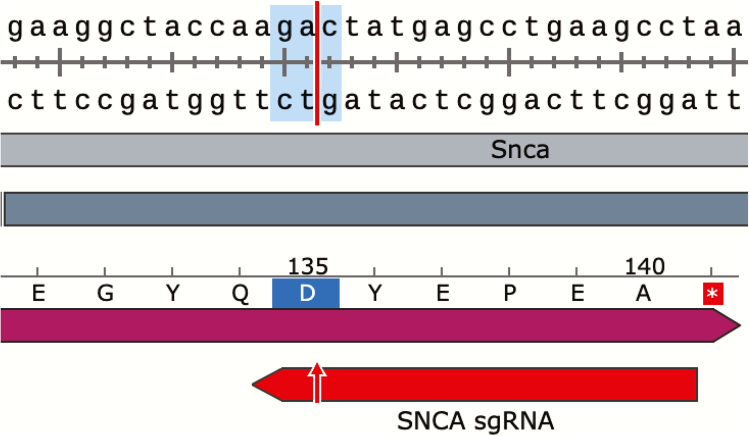
ORF at the Cas9 cleavage site for in-frame *Snca* CRISPR KI design An example of ORF +2 cleavage can be seen in the *Snca* gene, where the cut occurs within the codon for the amino acid 'D,' separating the sequence 'ga' from 'c.'.
    Follow the next steps for cloning and transformation.
  g. Prepare the reaction mix to digest 1 μg of the empty plasmid vector using BbsI HF, ensuring precise cleavage for downstream applications. Incubate the mixture at 37°C for 120 min to achieve complete digestion.

**Table:** 

1 ug	AAV α-syn:oScarlet KI donor
1 μL	BbsI HF (NEB)
3 μL	10x rCutSmart buffer
Up to 30 μL	ddH_2_O
30 μL	Total
  h. Run the digested DNA on a 1% agarose gel, excise and purify the desired band, then measure the plasmid concentration using a Nanodrop. Adjust the concentration to 10 ng/μL for subsequent applications.
    ***Note:*** The digested plasmid can be stored in a freezer and reused multiple times, withstanding repeated freeze-thaw cycles.
  i. Prepare the oligos for phosphorylation and annealing by assembling the following mixture:

**Table:** 

1 μL	*Snca*_KI_F (100 μM)
1 μL	*Snca*_KI_R (100 μM)
1 μL	10x T4 Ligation BF (NEB)
0.5 μL	T4 PNK (NEB)
6.5 μL	ddH_2_O
10 μL	Total
    Subsequently, program the thermocycler with the following steps.
    i. 37°C for 30 min to facilitate the enzymatic reaction.
    ii. 95°C for 5 min to denature the oligos.
    iii. Ramp down from 90°C to 25°C, decreasing by 5°C per minute, to allow gradual annealing.
    iv. Hold at 25°C to maintain stability for downstream use.
  j. Use the Annealed Oligos for Ligation and Transformation as described next:
    i. Prepare the ligation reaction:

**Table:** 

1 μL	BbsI digested plasmid (10 ng/ul)
0.5 μL	phosphorylated and annealed oligo duplex (1:100 dilution)
1.5 μL	2x Ligation Mix (Takara, Cat#6023)
3 μL	Total

      ***Note:*** The 1:100 dilution of the annealed oligo duplex reduces the risk of excess insert driving concatemer formation or reducing ligation efficiency. Add 0.5 μL of this diluted duplex directly into the ligation reaction.
    ii. Incubate the ligation reaction at 20-25°C for 15 min to allow efficient DNA ligation, then promptly place the mixture on ice to halt the reaction. Meanwhile, thaw a tube of One Shot Stbl3 Chemically Competent *E. coli* (Invitrogen, Cat#C737303) on ice for 5–10 min.
    iii. Add 10–15 μL of competent cells (One Shot Stbl3) to the ligation reaction, ensuring thorough mixing. Incubate the mixture on ice for 10–30 min to allow the DNA to properly bind to the cells while preserving their competency.
    iv. Heat shock the mixture at 42°C for 30 s to facilitate DNA uptake by the competent cells, then immediately place it on ice for 5 min to stabilize the cells and enhance transformation efficiency. After cooling, add 100 μL of SOC medium to the cells and incubate at 37°C with shaking at 225 rpm for 1 h.
    v. Plate the entire transformation mixture onto an LB agar plate containing ampicillin (100 μg/mL). Use plates pre-equilibrated to 20-25°C to promote optimal colony formation. Incubate plates 16-18 h at 37°C.
    vi. The following day, examine the plates. Typically, >95% of colonies should contain the correct sgRNA insert.
    vii. Pick individual colonies and inoculate into LB broth supplemented with ampicillin. Incubate 16-18 h at 37°C with shaking.
    viii. Isolate plasmid DNA from 16-18 h cultures using the QIAprep Spin Miniprep Kit (QIAGEN, Cat#27104).
    ix. Submit purified plasmid DNA for Sanger sequencing to confirm the presence and accuracy of the sgRNA insert.
    x. Once positive clones are verified, prepare transfection-grade plasmid DNA using the PureLink HiPure Plasmid Maxiprep Kit (Thermo Fisher Scientific, Cat#K210006).

### AAV production

**Timing: 1 week**

This section outlines a detailed protocol for AAV production using PEI Max transfection (Polysciences, Cat# 24765) in HEK293T cells, providing a rapid and cost-effective method for generating high-titer viral vectors suitable for experimental use. By optimizing parameters such as cell plating density, transfection reagent ratios, and harvest timing, this protocol reliably yields AAV preparations with titers typically between 1 × 10^12^ to 1.5 × 10^12^ viral genomes (vg)/mL.

4. Coating:
  a. Coat the plates or dishes with 0.01% PDL for at least 10 min at 20°C–25°C.
    For 12-well plates, use 2 mL of PDL per well; for 10-cm dishes, use 10 mL per dish.
    ***Note:*** The choice between 12-well plates and 10-cm dishes depends on the desired yield of AAV—use 12-well plates for small-scale production and 10-cm dishes for higher yields.
  b. After coating, rinse the plates or dishes once with 1X PBS to remove excess PDL.
5. Cell Plating:
  a. Seed HEK293T cells 24 h prior to transfection. For the 12-well plate format, seed 3 × 10^5^ cells per well. For the 10-cm dish format, plate 4.2 × 10^6^ cells per dish.

***Note:*** This protocol typically yields approximately 0.5–1 × 10^12^ viral genomes (vg)/mL, depending on the format used.

6. Transfection:
  a. In a separate tube, prepare the transfection mix by combining Opti-MEM with PEI Max, then add the plasmids according to the two tables below.
    **Table:** AAV-SaCas9-α-syn-KO

	12-well plate	10-cm dish
# of cells plated	3x 10^5^ cells/well	4.2x 10^6^ cells/dish
pHelper (Agilent Technologies, Cat # 240071)	0.49 μg	6.9 μg
PHP.eB (Addgene, plasmid #103005)	0.36 μg	5.1 μg
AAV SaCas9-α-syn-KO	0.22 μg	3.1 μg
Opti-MEM (Thermo Fisher Scientific, Cat# 31985062)	65 μL	900 μL
PEI Max (Polysciences, Cat#24765) (1 mg/mL)	4 μL	60 μL
Total volume	72 μL	963 μL

Note: To produce a control AAV, substitute the AAV SaCas9-α-syn-KO plasmid with the AAV SaCas9-Scramble-control plasmid.

**Table:** AAV-α-syn:oScarlet-KI

	12-well plate	10-cm dish
# of cells plated	3x 10^5^ cells/well	4.2x 10^6^ cells/dish
pHelper (Agilent Technologies, Cat # 240071)	0.49 μg	6.9 μg
PHP.eB (Addgene, plasmid #103005)	0.36 μg	5.1 μg
AAV α-syn:oScarlet-KI-donor	0.22 μg	3.1 μg
Opti-MEM (Thermo Fisher Scientific, Cat# 31985062)	65 μL	900 μL
PEI Max (Polysciences, Cat#24765) (1 mg/mL)	4 μL	60 μL
Total volume	72 μL	963 μL

Note: To produce the SpCas9 AAV, substitute the AAV α-syn:oScarlet-KI-donor plasmid with the AAV SpCas9 plasmid.
  b. Cap the tube securely and vortex for 5 s to ensure thorough mixing and homogeneity of the transfection mixture. Incubate the mixture at 20-25°C for 10 min to allow complex formation.
  c. Add the transfection solution dropwise to each well or dish, ensuring even distribution, then gently swirl the plates to facilitate proper mixing. Immediately return the plates to the incubator.
  d. Approximately 16–20 h post-transfection, replace the medium with fresh, pre-warmed DMEM+Glutamax/10% FBS /1% pen-strep media to support continued cell growth and expression.
7. AAV isolation:
  a. Two to four days after the medium change (typically after two days), carefully pipette the culture medium to detach the AAV-producing cells.
    ***Note:*** Ensure gentle handling, as cells usually detach easily in warm culture medium. This process is optimal around 2 days post-medium change but may vary slightly depending on cell conditions.
  b. Collect the detached cells into tubes and centrifuge at 2,000g for 5 min at 20°C–25°C. Carefully discard the supernatant without disturbing the cell pellet.
  c. Centrifuge the cell pellet again at 2,000g for 1 min and carefully remove all supernatant. Gently loosen the pellet by tapping the tube and vortexing briefly to ensure the cells are resuspended evenly.
  d. Add citrate buffer (see recipe in the “materials and equipment” section) (e.g., 100 μL per well for a 12-well plate or 1,000 μL per 10-cm dish), adjusting the volume based on the size of the plate or dish.
  e. Resuspend the cells thoroughly by tapping the tube gently and vortexing to ensure an even distribution.
  f. Incubate the sample at 37°C for 5 min, then vortex for 15 s.
  g. Centrifuge at 10,000g for 10 min at 4°C to separate the supernatant.
  h. Collect the supernatant into a sterile tube and add 1/10 volume of 2 M Tris-HCl (pH 9.5) to neutralize the solution.
  i. Gently mix to resuspend, then aliquot 100 μL into sterile Eppendorf tubes. Measure the viral titer by quantitative PCR (qPCR) and store the AAV preparations at −80°C until use.
    ***Note:*** In our experience, AAV preparations typically yield titers between 1x10^e12^ to 1.5x10^e12^ VG/ml. Vector packaging can be outsourced to commercial providers such as VectorBuilder (selecting the “Pilot Scale” option and specifying the AAV-PHP.eB serotype), although outsourcing typically requires 3–4 weeks for production and delivery. The resulting AAV preparations are ideal for downstream applications, including CRISPR-based gene editing, gene KOs, and other genetic manipulations in vitro or in vivo.

### Preparing hippocampal cultures for CRISPR editing experiments

**Timing: ∼2 h**

**Timing: 80 min (for step 8)**

**Timing: 30–45 min (for step 9a)**

**Timing: ∼15 min (for step 9b)**

**Timing: ∼5 min (for step 9c)**

**Timing: ∼5 min (for step 9d)**

**Timing: ∼25 min (for step 9e)**

This section describes the essential steps for preparing high-quality primary hippocampal neuron cultures, which form the foundation for successful CRISPR editing experiments. It details procedures for coating culture surfaces, dissecting and dissociating hippocampal tissue from neonatal mice, and plating neurons under sterile and optimized conditions. By emphasizing best practices—such as careful tissue handling, stringent sterility, and proper media preparation—this step ensures neuronal health and consistency, enabling efficient viral transduction, gene editing, and downstream functional analyses.

8. Coating
  a. Prepare a 1 mg/mL Poly-D-Lysine Hydrobromide (PDL) solution by dissolving PDL (Sigma, Cat# P6407-5MG) in Borax buffer, composed of 51.4 mM boric acid (Sigma, Cat# B6768) and 23.6 mM Borax (Sigma, Cat# S9640) in cell culture-grade H_2_O (Millipore, Cat# 4.86505), adjusted to pH 8.5.
  b. Filter-sterilize the solution using a 0.22 μm syringe filter.
  c. For imaging experiments:
    i. Sterilize an 18 mm round coverslip (Fisher Scientific, Cat# 50-948-975) by rinsing with 99% ethanol (Sigma, Cat# E7023-500ML), then flame-dry it using a Bunsen burner or alcohol lamp.
    ii. Place one sterile coverslip into each well of a 12-well plate.
    iii. Pipette 10 μL of the PDL solution directly onto the center of each coverslip, ensuring the droplet stays localized and does not spread across the surface.
  d. For biochemistry experiments (no coverslips needed):
    i. Add 500 μL of the PDL solution to each well of a 12-well plate, ensuring the entire surface is evenly coated.
  e. Incubate plates (with or without coverslips) at 20°C–25°C for 1 h.
  f. After incubation, wash wells at least 6 times with deionized distilled water (ddH_2_O) to remove excess PDL.
    **CRITICAL:** Incomplete washing with ddH_2_O will decrease neuron health and viability.
  g. Thoroughly aspirate any remaining water to dry the coverslips and let them sit in the biological safety cabinet (hood) until neurons are ready to be plated.
9. Dissection
  a. Immediately after rapid decapitation, dissect the hippocampi from P0-P1 mouse pups and transfer them into a 15 mL conical tube containing 5 mL of ice-cold HBSS buffer (Gibco, Cat#14025092).
    **CRITICAL:** During hippocampus dissection, carefully remove the fimbriae, a bundle of fibers that cover the medial edge of the hippocampus. Failure to do so can cause hippocampi to aggregate during dissociation, reducing neuronal yield. For a detailed isolation procedure and reference images, please refer to Cramer and Tyagarajan 2024^21^
  b. Digest hippocampi with Trypsin-EDTA:
    i. Transfer the desired number of dissected hippocampi from the ice-cold HBSS into a 15 mL conical tube containing 5 mL of pre-warmed 0.25% Trypsin-EDTA (Gibco, Cat# 25-200-072) at 37°C.
    ii. Incubate the tube in a 37°C water bath for 15 min to allow enzymatic digestion.
    iii. After incubation, terminate the digestion by transferring the hippocampi into 5 mL of blocking buffer in a separate 15 mL conical tube.
      ***Note:*** The blocking buffer should consist of 30% FBS and 70% 1× PBS, and be sterilized using a 0.22 μm syringe filter.
  c. Transfer hippocampi to plating media:
    i. Prepare 1 mL of plating media in a 15 mL conical tube. Use 10% FBS and 90% Neurobasal (Gibco, Cat# 21103049) supplemented with B27 (Gibco, Cat# 17504044), penicillin-streptomycin (Gibco, Cat# 15140122), and GlutaMAX (Thermo Fisher Scientific, Cat# 35050061).
    ii. Sterilize the complete media using a 0.22 μm syringe filter.
    iii. Gently transfer the hippocampi from the blocking solution into the conical tube containing the sterilized plating media (see detailed recipe in the “materials and equipment” section).
  d. Dissociate and count hippocampal cells:
    i. Triturate the hippocampi in 1 mL of plating media using a sterile P1000 pipette tip. Perform exactly 8 gentle passes to dissociate the tissue.
    ii. Observe the suspension and ensure it appears turbid with no visible tissue chunks remaining.
    iii. Pass the cell suspension through a 70 μm cell strainer (Corning, Cat# 431751) into a clean collection tube to remove debris.
    iv. Collect the filtered cell suspension and proceed to count the cells using a TC20 Automated Cell Counter (Bio-Rad, Cat# 1450102).
      ***Note:*** Use a hemocytometer or any other validated automated cell counting system, if preferred.
      **CRITICAL:** Avoid strong trituration and bubble-formation, which may cause cell death.
  e. Plate neurons and prepare for viral transduction:
    i. For imaging experiments, plate 30,000 cells in a 10 μL volume directly onto the center of each PDL-coated coverslip in a 12-well plate.
    ii. For biochemical experiments, plate 300,000 cells in 500 μL of media per well of a PDL-coated 12-well plate.
    iii. Place the plates in an incubator set to 37°C with 5% CO_2_ and allow the cells to recover for 20 min.
    iv. After recovery, gently add 1.5 mL of NB/B27 media to each well or dish without disturbing the plated cells.
    v. Return the plates to the incubator and maintain under standard culture conditions.
    vi. For AAV transduction, apply the virus 2–5 h after plating, following the procedure outlined in the AAV transduction section for CRISPR KO or KI experiments.
    vii. If using lentiviruses for CRISPR KO, transduce hippocampal neurons at DIV3 as described in the lentivirus packaging production and transduction for CRISPR KO section.
    viii. Maintain the cultures in NB/B27 media until neurons reach maturity at DIV21.
      ***Optional:*** Replace half of the media (1 mL) with fresh NB/B27 media (see detailed recipe in the “materials and equipment” section) every 7 days.

### AAV transduction

**Timing: 48 h**

This section describes a protocol for AAV transduction of cultured hippocampal neurons to deliver CRISPR/Cas9 components for gene KO or KI experiments. By controlling plating density, multiplicity of infection (MOI), and timing, this procedure ensures high transduction efficiency while minimizing cytotoxicity. The protocol provides instructions for both immunofluorescence-based and biochemical assays, tailoring AAV volumes and formats to different experimental needs. After a 48-h transduction period, neurons are thoroughly washed to remove residual virus and returned to conditioned media to promote healthy recovery and continued culture. This method ensures reliable gene editing outcomes while preserving neuronal viability and morphology.

10. Cell Plating:
  a. For immunofluorescence experiments, plate 30,000 hippocampal neurons (in ∼10 μL volume) onto the center of each PDL-coated coverslip placed in a 12-well plate.
  b. For biochemical experiments, plate 300,000 hippocampal neurons per well directly onto PDL-coated wells of a 12-well plate.
  c. Allow cells to recover in a 37°C, 5% CO_2_ incubator for 20 min. After recovery, gently add 2 mL of complete Neurobasal/B27 (NB/B27) medium to each well.
11. Initial Incubation and Virus Thawing:
  a. Incubate neurons at 37°C with 5% CO_2_ for 2–5 h to allow for cell attachment and stabilization.
  b. During this time, thaw AAV stocks (SaCas9 for CRISPR KO; SpCas9 and KI donor for CRISPR KI) on ice from −80°C storage.
12. Preparation of AAV transduction mix:
  a. For biochemical CRISPR KO experiments, prepare the AAV transduction mix (scramble control or α-synuclein CRISPR KO) by diluting the appropriate volume of virus (calculated to achieve a final MOI of 100,000) into pre-warmed NB/B27 medium for a total volume of 500 μL.
  b. For immunofluorescence CRISPR KI experiments, prepare the AAV transduction mix by diluting the appropriate volumes of AAV SpCas9 and AAV α-syn:oScarlet KI donor into pre-warmed NB/B27 medium to achieve a combined MOI of 100,000 (i.e., 50,000 MOI per virus) in a final volume of 200 μL.

***Note:*** Adjust the volume of each viral preparation based on its individual titer (vg/μL) to accurately achieve the desired MOI relative to the plated cell density.

***Note:*** The KI strategy for inserting a fluorescent Scarlet tag at the C-terminus of endogenous mouse α-syn requires co-transduction with two separate AAVs: the AAV α-syn:oScarlet KI donor and AAV-SpCas9. Genome editing occurs only in neurons that receive both vectors, making the approach inherently more challenging and less efficient due to the requirement for dual infection. To optimize transduction efficiency, adjust the volume of each AAV preparation according to its viral genome titer (vg/μL) to achieve the appropriate MOI based on the plated cell density.

13. Equilibration and Media collection:
  a. Incubate the prepared AAV mix at 37°C for 10 min to equilibrate.
  b. Carefully remove 700 μL of conditioned medium from each well and transfer it to a sterile 15 mL Falcon tube.
  c. Maintain sterility and keep the collected medium warm at 37°C for later use.
14. AAV Transduction and incubation:
  a. Slowly add 500 μL (for biochemical experiments) or 200 μL (for immunofluorescence experiments) of the AAV mix directly to the neurons, taking care not to disturb the cells.
  b. Return the 12-well plate to the 37°C, 5% CO_2_ incubator and incubate for 48 h.

***Note:*** Prolonged incubation beyond 48 h may lead to increased cytotoxicity.

15. Post-Transduction Washing and Recovering:
  a. Following the 48-h incubation, wash off the virus by adding 1 mL of fresh NB/B27 medium and removing 1 mL of existing media.
  b. Repeat the wash step four times, using gentle pipetting to minimize mechanical stress on the neurons.
  c. After the final wash, add back the saved 700 μL of conditioned medium to each well.
  d. Return the plate to the 37°C incubator for continued culture, typically until neurons reach the desired maturity.

### Lentivirus packaging production and transduction for CRISPR KO

**Timing: 1 week (for step 16)**

**Timing: 6 h (for step 17)**

This section provides an alternative strategy for CRISPR-mediated KO of α-syn in cultured hippocampal neurons using lentiviral vectors. Lentiviruses offer stable integration of CRISPR components into the neuronal genome, providing a reliable and efficient method for long-term gene disruption. The protocol describes the complete process, from production of high-titer lentiviruses in HEK293T cells to neuronal transduction and post-infection recovery. Special attention is given to optimal viral titers, careful removal of residual lentivirus after transduction, and minimizing mechanical stress to preserve neuronal viability. This approach is particularly useful for experiments requiring sustained CRISPR expression and is a valuable alternative when AAV-mediated CRISPR delivery is not optimal.

16. Lentivirus production:
  a. Before beginning lentivirus production, maintain HEK293T cells in DMEM+Glutamax (Gibco, Cat#10566016) supplemented with 10% FBS and 1% pen-strep (Gibco, Cat#15140122) (5% CO_2_, 37°C).
    ***Note:*** HEK293T cells should be thawed and passaged at least two times before plating.
  b. Prepare PDL solution (100 μg/mL in water, sterilized by filtration). To coat dishes, add 20 ml of the PDL solution to each 15 cm cell culture dish (8–10 dishes total) and incubate at 20°C–25°C for 30 min.
  c. Wash the dishes three times with cell culture-grade water and allow them to air-dry completely at 20°C–25°C.
  d. The day before transfection, seed 6x10^6^ HEK293T cells in each 15 cm dish with 20 ml of DMEM+Glutamax/10% FBS /1% pen-strep media. Cells should reach 70-80% confluency within 14-16 h.
  e. Equilibrate ProFection transfection reagent kit (Promega, Cat#E1200) to 20°C–25°C and mix thoroughly.
  f. In a Falcon tube, combine the following components to prepare the DNA mix:

**Table:** 

Component	Amount
Targeting plasmid (*LentiCRISPR v.2_Scramble Control* or *LentiCRISPR v.2_α-syn KO*)	20.4 μg
psPAX2 packaging plasmid (Addgene #12260)	15.6 μg
pMD2.G envelope plasmid (Addgene #12259)	10.2 μg
CaCl_2_ solution (from ProFection kit)	93 μL
Sterile water	X μL to reach the final volume
Final volume	750 μL
  g. Vortex the DNA mix for 5–10 s to ensure complete homogenization.
  h. Using a P1000 pipette, slowly add the DNA mix dropwise to a second tube containing 750 μL of 2x HEPES-buffered saline (HBS) solution (provided in the ProFection kit), while continuously vortexing to ensure thorough mixing.
  i. Incubate the solution at 20-25°C for 30 min to allow precipitate formation.
  j. Replace the media from the HEK293T cells with 20 ml pre-warmed fresh DMEM+Glutamax/10% FBS /1% pen-strep media.
  k. After the 30-min incubation, vortex DNA-HBS mix again and add the solution dropwise to the HEK293T cells, swirling the dish to ensure even distribution. Place cells in the incubator 16–18 h.
    ***Caution:*** All work involving lentiviral vectors must be performed in accordance with institutional biosafety regulations and approved by the appropriate biosafety committee. Follow Biosafety Level 2 (BSL-2) guidelines. All materials that come into contact with lentivirus must be disinfected by soaking in 10% bleach for at least 30 min prior to disposal (as per UC San Diego laboratory safety guidelines).
  l. Perform Media Change Post-Transfection: 12–16 h after transfection, aspirate the media to remove transfection reagents. Replace with 20 mL of fresh pre-warmed DMEM+Glutamax/10% FBS /1% pen-strep media.
  m. Harvest Viral Supernatant: 24 h later, collect the media containing lentiviral particles into 50 mL Falcon tubes and store at 4°C. Add 20 mL of fresh pre-warmed DMEM+Glutamax/10% FBS /1% pen-strep media to the cells and incubate 16–18 h.
  n. After another 24 h (48 h post-transfection), collect the DMEM+Glutamax/10% FBS /1% pen-strep media again and pool with the previous collection.
  o. To clarify the viral supernatant centrifugate the pooled media at 500xg for 10 min at 4°C to pellet any cell debris.
  p. Carefully transfer 36 mL of the supernatant to a new 50 ml Falcon tube and add 12 ml of Lenti-X (Takara, Cat#631232).
    **CRITICAL:** When collecting the 36 ml supernatant, avoid disturbing the cell debris pellet to prevent contamination of the viral supernatant, which can reduce viral yield and purity. To minimize disruption, tilt the 50 mL Falcon tube slightly and gently take the media from the edge of the tube using a serological pipette. Keep the pipette tip above the pellet—do not touch or disturb the debris.
  q. Incubate the tubes at 4°C on a laboratory rocker for at least 4 h.
    ***Optional:*** Incubation can be extended to 12–16 h without affecting titers.
  r. Centrifugate the 50 ml falcon tubes at 1500xg for 45 min at 4°C.
  s. Carefully discard the supernatant.
    ***Note:*** We recommend tilting the tube and gently aspirating the supernatant using a 25 mL serological pipette.
  t. Add 100 μL of ice-cold, filtered HBSS to each viral pellet.
  u. Gently resuspend the pellet using a P1000 pipette, avoiding bubble formation.
  v. Combine the resuspended virus from all tubes into a single sterile tube and mix gently by pipetting up and down.
  w. Aliquot 100 μL of the virus into sterile microcentrifuge tubes.
    ***Note:*** Small aliquot volumes are recommended to minimize freeze-thaw cycles, which can significantly reduce viral titer.
  x. Store aliquots at −80°C for long-term storage or at −20°C for short-term use (up to 1 week).
    **CRITICAL:** Avoid generating bubbles during resuspension, as this can compromise viral integrity and significantly reduce viral titers.
  y. Thaw lentivirus on ice prior to titering, as viral titers decrease rapidly at elevated temperatures.
  z. Determine lentivirus titer using Lenti-X GoStix (Takara, Cat#631280) according to the manufacturer’s instructions (https://www.takarabio.com/documents/User%20Manual/Lenti-X%20GoStix%20Plus%20Protocol-At-A-Glance_021121.pdf), or alternatively, use a qPCR-based titration method.
    ***Note:*** In our experience, lentiviral preparations typically yield titers ranging from 1x10^e7^ to 2x10^e7^ particles/ml.
17. CRISPR KO lentiviral transduction.
  a. Thaw the vial of lentivirus on ice prior to neuronal transduction.
  b. Calculate the amount of lentivirus needed using a MOI of 5. For 30,000 neurons, MOI=5 requires 150,000 viral particles. For example, if the viral titer is 1x10^e7^ particles/ml, use the following formula to calculate the volume:
    Titer x Volume = Number of particles.
    1x10 ^e7^ particles/ml x Volume = 150,000 particles.
    Volume =0.015 ml=15 μL
    ***Note:*** Hippocampal cultures can tolerate MOI of up to 8; higher MOIs can cause increased cell death.
  c. Dilute X μL of lentivirus (Lenti SpCas9 Scramble Control or Lenti SpCas9 α-syn KO) in NB/B27 medium, adjusting the volume based on viral titer, to a final volume of 200 μL.
  d. Incubate the virus mix at 37°C for 10 min before adding it to the neurons.
  e. Remove 1 mL of conditioned medium from each well of the 12 well-plate (with or without coverslips) and transfer it to a sterile 15 ml conical tube for later use.
  f. Slowly add the 200 μL lentivirus mix on top of the neurons, being careful not to disturb them. Return the plate to the incubator (5% CO_2_, 37°C) for 6 h.
    ***Note:*** Longer incubation times can lead to cell death.
  g. After incubation, wash off the lentivirus by adding 1 ml of fresh NB/B27 medium to the culture, then carefully remove 1 ml of the media. Repeat this wash step four times, ensuring gentle handling during each step.
  h. After the final wash, add the previously saved 1 mL conditioned NB/B27 medium back to each well of the 12-well plate. Return the plate to the 37°C incubator and continue culturing the neurons, typically until they reach synaptic maturity (DIV17–21).
    ***Optional:*** Replace 1 mL of medium with fresh NB/B27 every 7 days.
    ***Note:*** Follow biosafety guidelines when working with lentiviral vectors, including using appropriate personal protective equipment (PPE) and working in a BSL-2 biosafety cabinet.

### Validation of CRISPR KO and KI by western blotting and immunofluorescence

**Timing: ∼3 days**

This step outlines the procedures for validating CRISPR-mediated KO and KI of α-syn in cultured hippocampal neurons. Accurate validation is critical to confirm successful genome editing and to assess editing efficiency. Western blotting is employed to detect changes in α-syn protein levels following KO or KI, using appropriate controls to ensure specificity. In parallel, immunofluorescence analysis enables visualization of α-syn localization, CRISPR components, and KI reporters at the single-cell and synaptic level. Detailed quantification using MetaMorph software ensures robust and reproducible analysis of editing outcomes. Together, these complementary approaches provide a comprehensive validation framework, confirming both the biochemical and cellular effects of CRISPR editing in neuronal cultures.

18. Western blotting Validation.
  a. Wash 300,000 neurons (DIV 17-21), previously transduced with AAVs for α-syn KO or KI, with 1 mL of 1X PBS.
    ***Note:*** For CRISPR KO experiments, use neurons infected with scramble control AAV. For KI experiments, use neurons infected with the α-synuclein KI AAV alone (no SpCas9) as the control.
  b. Aspirate the 1X PBS completely and immediately lyse the neurons by incubating them with 100-150 μL of neuronal lysis buffer (buffer recipe in “Materials and Equipment” section), for 5-10 min on ice.
  c. Centrifuge the lysates at 10,000 × g for 10 min at 4°C.
    ***Note:*** Carefully transfer the resulting supernatant—which contains the solubilized proteins—to a new 1.7 mL Eppendorf tube, taking care not to disturb the pellet of cellular debris. We recommend tilting the tube and gently aspirating the supernatant using a P1000 pipette for optimal recovery.
  d. Determine the total protein concentration of the cleared lysates using the DC Protein Assay Kit II (Bio-Rad, Cat#5000111) (https://www.bio-rad.com/webroot/web/pdf/lsr/literature/LIT448.pdf), following the manufacturer’s instructions.
  e. Mix the appropriate volume of lysate with 4X NuPAGE LDS sample buffer (Thermo Fisher Scientific, Cat#NP007) to achieve the desired final concentration (typically 1 μg/μL). We typically load between 10-20 μg of protein for western blots.
  f. Heat the mixture at 95°C for 10 min to denature the proteins.
  g. Assemble the gel tank incorporating the gel of choice. In our case, we use the NuPAGE 4 to 12% Bis-Tris polyacrylamide gel (Thermo Fisher Scientific, Cat#NP0335BOX).
  h. Fill the inner and outer chambers of the tank with 1X running buffer (see buffer recipe in the “materials and equipment” section).
  i. Load 10–20 μg of protein per lane on the NuPAGE gel.
    ***Note:*** We recommend loading a final volume of 10–50 μL per well, depending on protein concentration.
  j. Run the gel at 80 V for 1.5–2 h or until the dye front reaches the bottom and runs off the gel.
  k. Transfer the gel to a 0.2 μM PVDF membrane (Thermo Fisher Scientific, Cat#LC2002), using 1X transfer buffer (refer to recipe details listed under ‘‘materials and equipment’). Transfer at constant 30 V for 90 min using the Mini Blot Module system (Thermo Fisher Scientific).
  l. After complete transfer, rinse the membrane twice with 1X PBS.
  m. Fix the membrane in 0.2% PFA diluted in 1X PBS for 30 min at 20-25°C, gently rotating to ensure even fixation across the membrane surface.
    ***Note:*** The fixation step ensure retention of α-synuclein on the membrane post-transfer.
  n. Rinse PFA by incubating the membrane in wash buffer containing PBS with 0.1% Tween 20 Detergent (PBST). Perform the wash for 30 min, changing the buffer every 10 mins.
  o. Block the membrane in 5% milk prepared in PBST buffer (blocking solution) for 1 h at 20°C–25°C with gentle agitation.
  p. Prepare the primary antibody solution by diluting the mouse monoclonal anti-α-synuclein antibody (BD Bioscience, Cat# 610787, RRID:AB_398108) in blocking buffer (see "key resources table" for dilution details).
    ***Note:*** Additional primary antibodies (e.g., anti-β-synuclein, Synapsin I, VAMP2, Complexin1/2, SpCas9, GAPDH) may be used as controls. See “key resources table” for antibody details and recommended dilutions.
  q. Following the blocking step, incubate the membrane with the mouse monoclonal anti-α-synuclein primary antibody (BD Bioscience, Cat# 610787, RRID:AB_398108) diluted in blocking solution. Incubate for 12–16 h at 4°C with gentle agitation to ensure even antibody binding.
  r. The next day, wash the membrane 3 times with PBST (10 min each wash) using a rotator.
  s. Incubate the membrane with HRP-conjugated goat anti-mouse secondary antibody (Abcam, Cat#ab205719) diluted 1:2000 in blocking buffer for 1 h at 20°C–25°C with gentle agitation (see “key resource table” for antibody details).
  t. Wash the membrane 3 times with PBST (10 min each) on a rotator.
  u. Detect HRP activity using enhanced chemiluminescence (ECL) substrate. We routinely use SuperSignal West Femto Maximum Sensitivity Substrate (Thermo Fisher Scientific, Cat#FER34095X4).
  v. Visualize protein bands using ChemiDoc Imaging System (Bio-Rad) and quantify data using Image Lab software (version 6.1 from Bio-Rad, RRID:SCR_014210).
    ***Note:*** To re-probe the membrane with a different antibody, incubate the membrane in stripping buffer for 20 min at 37°C with gentle shaking. In this protocol, we used Restore Plus Western Blot Stripping Buffer (Thermo Fisher Scientific, Cat# 46430). Following stripping, wash the membrane three times for 10 min each with PBST on a rotator. Subsequently, block the membrane for 1 h at 20°C–25°C, then proceed with immunostaining by incubating with the new primary antibody as described above.
    ***Caution:*** Repeated membrane stripping more than twice can result in reduced protein detection.
19. Immunofluorescence Validation.
  a. In the 12-well plate containing gRNA/Cas9-treated neurons on coverslips, carefully wash the cells twice with 1X PBS/sucrose buffer (see recipe in the “materials and equipment” section under “Buffers for Immunocytochemistry in Neurons”) to remove cellular debris.
  b. Aspirate the PBS/sucrose buffer using a P1000 pipette and fix the neurons by adding 500 μL of fixation buffer (see “materials and equipment” section under “Buffers for Immunocytochemistry in Neurons”) per well. Incubate for 10 min at 20°C–25°C.
  c. Remove the fixation buffer with a P1000 pipette and wash the neurons three times with 1 mL of 1X PBS per well (aspirating and replenishing PBS between washes).
  d. Permeabilize the neurons by incubating them in 500 μl of extraction buffer (see recipe in the “materials and equipment” section under “Buffers for Immunocytochemistry in Neurons”) for 10 min at 20-25°C.
  e. Wash fixed/permeabilized neurons with 1X PBS three times.
  f. Block non-specific binding sites by incubating the neurons in blocking buffer (refer to recipe details listed under “Buffers for Immunocytochemistry in Neurons” in the ‘‘materials and equipment’’ Section) for 2 h at 20°C–25°C.
  g. Incubate the neurons 16–18 h at 4°C with primary antibodies diluted 1:500 in 1× PBS.
    ***Note:*** For CRISPR KO experiments, use mouse monoclonal anti-α-synuclein antibody (Synaptic Systems, Cat#128211, RRID:AB_2619811) and rabbit polyclonal anti-SpCas9 antibody (Cell Signaling Technology, Cat#19526, RRID:AB_2798820); staining for SpCas9 is critical to assess AAV transduction efficiency and genome editing. For CRISPR KI experiments, stain hippocampal cultures with mouse monoclonal anti-VGLUT1 antibody (Synaptic Systems, Cat#135303, RRID:AB_887875) at the same dilution.
  h. The next day, wash neurons 3 times with 1x PBS (5 min each).
  i. Block neurons again with blocking solution for 30 min at 20°C–25°C.
  j. Incubate the neurons with goat anti-mouse Alexa Fluor 488 (Thermo Fisher, RRID: AB_2633275) and goat anti-rabbit Alexa Fluor 594 (Thermo Fisher, RRID: AB_2762824) secondary antibodies, both diluted 1:500 in 1X PBS (500 μl final volume). Incubate for 1 h at 20°C–25°C in the dark to protect from light.
  k. Wash neurons 3 times with 1x PBS for 5 min each to remove excess secondary antibodies.
    **CRITICAL:** Ensure all incubations with secondary antibodies are done in the dark to avoid photobleaching.
  l. Mount the coverslips onto microscope slides using a fluorescence-compatible mounting medium. In this protocol, we used Fluoro-Gel (Electron Microscopy Sciences, Cat# 17985-11). Allow the mounting medium to cure at 20°C–25°C, then store the slides protected from light at 4°C until imaging.
  m. Acquire Z-stack images using a microscope at 40x magnification.^22^
  n. Use MetaMorph Microscopy Automation and Image Analysis Software (RRID:SCR_002368)) for image acquisition and processing (https://www.moleculardevices.com/products/cellular-imaging-systems/acquisition-and-analysis-software/metamorph-microscopy#gref). For quantification of the KI α-syn:oScarlet fluorescence at synapses, images were first background-corrected, small regions of interest (ROIs) were manually placed over ∼20-30 synapses on each image, and average intensities were calculated – all using dropdown menus (see below).
20. Immunofluorescence quantification using MetaMorph.
  a. Background Correction/Subtraction:
    i. Launch MetaMorph and open your raw image stack.
    ii. Navigate to the Process menu and select Background and Shading Correction.
    iii. Enable the Statistical Correction option.
    iv. Draw a region in an area of the image that does not contain any fluorescent signal (background region).
    v. Select all frames in the image stack.
    vi. Check the Average option and click Apply to perform background correction and obtain a statistically corrected image stack.
  b. Generate Maximally Projected Image:
    i. Select the statistically corrected image stack.
    ii. Go to the Process menu and choose Stack Arithmetic.
    iii. Check the Max option and click Apply to generate a maximally projected image.
  c. Thresholding and Region Creation:
    i. To identify fluorescent synapses, go to the Measure menu and select Threshold.
    ii. Use the Inclusive option to set the appropriate threshold values in the provided boxes.
    iii. Navigate to the Region menu and select Create Region Around Objects to outline regions around the thresholded fluorescent synapses.
    iv. Save the created regions.
  d. Transfer and Analyze Regions:
    i. Mark and save the regions corresponding to fluorescent synapses in the images from both channels.
    ii. Use the Edit menu and select Transfer Region to transfer regions from α-syn synapses to VGLUT1 synapses.
    iii. For transferring regions, go to the Edit menu and choose Transfer Region.
    iv. Ensure that only the regions overlaying fluorescent synapses are retained (VGLUT1-expressing cells). Delete any remaining areas.
    v. Calculate the fraction of synapses positive for AAV α-syn:oScarlet KI editing by dividing the number of overlapping regions by the total number of VGLUT1 synapses.
  e. Control Analysis:
    i. Perform parallel analyses on synapses treated with either AAV α-syn:oScarlet KI alone or AAV SpCas9 alone to assess the likelihood of leaky expression or false-positive signals.
  f. Measure Fluorescent Intensity:
    i. Load the saved regions onto the respective synapse images.
    ii. Go to the Measure menu and select Region Measurements to display the average fluorescent intensity of individual synapses.
    iii. Click Open Log to export the measurement data.
  g. Data Analysis:
    Measure and record the average fluorescence intensity for images corresponding to the following AAV transduction conditions.
    i. AAV α-syn:oScarlet KI donor + AAV SpCas9.
    ii. AAV α-syn:oScarlet KI donor only.
    iii. AAV SpCas9.
      This stepwise approach ensures accurate processing and quantification of fluorescent synapses, allowing for a thorough analysis of synapse expression and editing efficiency.

### Validation of CRISPR KO by Sanger sequencing and subsequent analysis using TIDE

**Timing: ∼7 days**

**Timing: 1 h (for step 21)**

**Timing: 7 days (for step 22)**

**Timing: 30 min (for step 23)**

The goal of this step is to validate the efficiency and specificity of CRISPR-mediated KO in hippocampal neurons by preparing genomic DNA, performing targeted amplification and Sanger sequencing, and analyzing the resulting data using TIDE. High-quality genomic DNA is first extracted from edited and control neurons to serve as a template for PCR amplification of both on- and predicted off-target sites. Carefully designed and validated primers ensure specific amplification of target regions, which are then subjected to Sanger sequencing. Sequencing traces are analyzed using TIDE to quantify the frequency and types of insertions and deletions (indels) introduced at the CRISPR target site, providing a robust measure of editing efficiency. Additionally, off-target analyses help assess the specificity of the CRISPR system. This validation step is essential for confirming successful genome editing and for optimizing CRISPR experimental conditions to achieve reliable, reproducible results.

21. Preparation of genomic DNA.

This section describes the preparation of genomic DNA from CRISPR-edited neurons for Sanger sequencing and subsequent TIDE analysis.

  a. Wash 300,000 cultured hippocampal neurons (DIV 17-21), previously infected with scrambled control or α-syn CRISPR KO lentivirus, with 1 mL of 1X PBS.
  b. After removing the 1X PBS add 50 μL of Lucigen QuickExtract DNA Extraction Solution (Biosearch Technologies, cat# QE09050) to each well/sample of neurons.
  c. Gently mix by pipetting up and down to ensure proper lysis of the cells.
  d. Transfer the mix to PCR tubes and use the thermocycler for incubation as follows:
    i. 68°C for 15 min.
    ii. 95°C for 10 min.
  e. After incubation, store the lysates at −20°C for future downstream analysis.

22. Sanger Sequencing.
  TIDE is a tool designed to quantify the frequency of insertions and deletions (indels) seen in Sanger sequencing resulting from non-homologous end joining (NHEJ) at nominated target sites. For our experiments, the Mus musculus genome assembly GRCm38 (mm10) is used as the reference genome, though this will vary depending on the relevant species. Ensure that the appropriate reference genome is selected to guarantee accurate alignment and mutation analysis.
  a. Identify potential off-target sites using CasOFFinder (http://www.rgenome.net/cas-offinder/).Select the appropriate reference genome and specify the PAM sequence for your CRISPR system. Input the guide RNA sequence for analysis.
    ***Note:*** The query parameters used for this analysis are as follows:
    Mismatch Number: 3.
    DNA Bulge Size: 0.
    RNA Bulge Size: 0.
    Off-target sites with more than 3 mismatches are generally considered unlikely to result in significant off-target effects, as supported by previous studies.^23^
  b. Design primers via SnapGene with a GC content of 40-60% and a length of 18-24 base pairs (bp) to amplify on- and off-target gRNA sites. Submit the primers to IDT for Sanger sequencing.
    ***Note:*** Primers for on- and off-target analysis were optimized to start and end with a C or G whenever possible for enhanced stability. The designed primers have similar melting temperatures to ensure uniform amplification and are optimized to amplify fragments of 1000-1500 bp from the target sequence. The designed Sanger sequencing primers are ∼170-300 bp upstream of the CRISPR cut site, in the direction of the gRNA, to ensure coverage of the target region for sequencing.
  c. Use the BLAST-like alignment tool (BLAT) to check the specificity of each primer. Primers should be specific to your region of interest (https://genome.ucsc.edu/cgibin/hgBlat?hgsid=2143016336_Uz9QWELjorobDmSjYGqjLthfwPaU&command=start).
    ***Note:*** Ensuring primer specificity is critical to avoid amplification of off-target or non-specific genomic regions. Poorly designed primers can result in ambiguous Sanger sequencing data and compromise the accuracy of downstream analyses, including TIDE quantification.
  d. Perform In-Silico PCR to simulate amplification and check if the designed primers may bind and amplify unintended genomic regions (https://genome.ucsc.edu/cgi-bin/hgPcr).
    ***Note:*** While this tool cannot guarantee the identification of all potential off-target amplicons, it provides a useful indication of whether the primers are likely to amplify non-specific products.
  e. Evaluate the potential for primer-dimer formation—both self-dimers and cross-dimers—using the Primer Analyzer tool (https://www.thermofisher.com/us/en/home/brands/thermo-scientific/molecular-biology/molecular-biology-learning-center/molecular-biology-resource-library/thermo-scientific-web-tools/multiple-primer-analyzer.html).
    ***Note:*** Minimizing primer dimer formation is crucial for efficient and accurate PCR amplification.
  f. Amplify gDNA from edited neurons and experimental controls using a high-fidelity DNA polymerase such as Q5 or Phusion.
    ***Note:*** Include gDNA from non-transduced neurons (negative control) and scrambled sgRNA-treated neurons (non-targeting control) alongside your edited samples to assess editing specificity and efficiency.
    i. Set up the reaction mix and PCR conditions using the manufacturer’s protocol (https://www.neb.com/en-us/protocols/0001/01/01/pcr-protocol-m0530). The final volume should be 50 μL to ensure sufficient product for PCR purification.
    ii. Prepare a master mix that includes all components except the DNA polymerase and primers. Use primers at a final concentration of 0.5 μM and add 1 μL of DNA polymerase. Start with 100 ng of gDNA per reaction to ensure robust amplification.
    iii. Perform 35 cycles for gDNA amplification.
  g. Verify successful amplification by running 4 μL of the PCR product mixed with 1 μL of 6X loading dye on a 1% agarose gel. Confirm that the resulting band is the expected size and that no additional, non-specific bands are present.
    ***Note:*** Unexpected bands may indicate off-target amplification. Before redesigning primers, PCR-purify the negative control samples and perform Sanger sequencing. If the chromatogram (.ab1 file) shows clean traces with no double peaks and aligns with the target sequence, proceed with positive controls. If multiple peaks are present, the sequencing primer may have bound to an unintended site. In this case, optimize the PCR conditions by increasing the annealing temperature to enhance specificity. If non-specific amplification persists after optimization, redesign primers targeting a different region and repeat the PCR.
  h. Purify PCR products using the QIAGEN QIAQuick PCR Purification Kit (Cat# 28104) following the manufacturer’s instructions (https://www.qiagen.com/us/resources/resourcedetail?id=e0fab087-ea52-4c16-b79f-c224bf760c39&lang=en).
  i. Elute the purified DNA in 20-30 μL of molecular-grade water to concentrate the sample for downstream sequencing.
    ***Note:*** If clean, specific bands cannot be obtained after multiple rounds of primer redesign and optimization, consider carefully excising and gel-purifying the band corresponding to the expected amplicon size. However, keep in mind that gel purification carries an increased risk of contamination and sample loss. Additionally, residual ethanol from the purification process can interfere with Sanger sequencing quality, leading to poor or noisy traces. Ensure thorough drying of the gel-purified DNA and follow best practices to minimize contamination before proceeding with sequencing.
  j. Submit your samples to Genewiz (Azenta Life Sciences) for Sanger sequencing using the purified PCR product service. For sequencing, use the previously designed Sanger sequencing primers.
    ***Note:*** Before proceeding with TIDE analysis, visually inspect the .ab1 chromatogram files from negative control samples (e.g., untransduced or scrambled-treated) using software such as SnapGene or Benchling. Confirm that the sequencing traces are clean, with well-defined single peaks and no evidence of insertions, deletions, or mutations at the target site. The presence of double peaks or sequence ambiguities in control samples may indicate off-target amplification or sequencing artifacts and should be resolved before analyzing edited samples.
23. TIDE analysis.
  a. Perform TIDE analysis using the TIDE Batch tool (http://shinyapps.datacurators.nl/tide-batch/) with default settings to quantify genome editing outcomes from Sanger sequencing data.
  b. Analyze both on- and off-target sites to determine the frequencies of insertions and deletions (indels). For off-target sites, use the mismatching guide RNA sequences identified by CasOFFinder.

### Validation of CRISPR KI by PCR

**Timing: ∼3 h**

**Timing: ∼30–45 min (for step 24)**

**Timing: ∼2 h (for step 25)**

The purpose of this step is to validate the successful CRISPR-mediated KI of an oScarlet tag into the target gene in hippocampal neurons. This step involves extracting genomic DNA from transduced neurons and performing PCR to amplify regions spanning the integration junctions of the inserted tag. By utilizing two independent PCR reactions, this approach enhances the sensitivity and accuracy of detecting successful KI events. PCR products are then analyzed through agarose gel electrophoresis to confirm the expected band sizes, with further verification through sequencing. This validation ensures the precise integration of the CRISPR edit and is critical for confirming the success of the KI strategy.

24. Genomic DNA extraction
  a. Add 50 μL of QuickExtract DNA Extraction Solution (Biosearch Technologies, Cat# QE09050) directly to each hippocampal neuron sample transduced with CRISPR KI components.
  b. Mix thoroughly by pipetting up and down to ensure complete cell lysis, then transfer the lysate into a PCR tube for thermal incubation.
  c. Place the samples in a thermocycler and incubate at 68°C for 15 min.
  d. Increase the temperature to 95°C and incubate for 10 min to inactivate enzymes.
  e. Store the extracted genomic DNA at −20°C until ready for use.
25. PCR KI validation.
  a. Set up two independent PCR reactions using the extracted genomic DNA to amplify genomic regions spanning the oScarlet integration junctions.
    ***Note:*** While a single PCR can be sufficient, we optimized two reactions to enhance detection sensitivity and ensure accurate validation. Each reaction is designed to amplify regions spanning the genomic integration junctions of the inserted oScarlet tag. This dual-PCR strategy enables robust confirmation of successful CRISPR-mediated integration. Set up the two separate PCR reactions using the extracted genomic DNA as template, as outlined below:
    PCR #1 (∼500 bp):
    Forward primer: TGTGCTTTCTCTTCCCTCTCTG (α-synuclein forward primer)
    Reverse primer: CCGTCCTCGAAGTTCATCAC (oScarlet reverse primer)
    PCR #2 (∼500 bp):
    Forward primer: ATAACACTTCGTGCAGCACC (α-synuclein forward primer)
    Reverse primer: ACAGGATGTCCCAGGAGAAG (oScarlet reverse primer)
    The table below outlines the PCR reaction mix and corresponding cycling conditions.
    PCR table
    **Table:** PCR reaction master mix

Reagent	Amount
DNA template (extracted DNA)	1 μl
Phusion DNA Polymerase	0.5 μl
Forward primer (10 μM)	1 μl
Reverse primer (10 μM)	1 μl
dNTPs (10 mM)	1 μl
5X Phusion HF buffer	1 μl
Nuclease-free water	44.5 μl

**Table:** PCR cycling conditions

Steps	Temperature	Time	Cycles
Initial Denaturation	98 °C	30 sec	1
Denaturation	98 °C	30 sec	30 cycles
Annealing	60 °C	30 sec	
Extension	72 °C	30 min	
Final extension	72 °C	5 min	1
Hold	4 °C	forever
  b. Prepare a 1% agarose gel by dissolving agarose in 1x TAE buffer.
  c. Pour the gel into a casting tray and insert a comb to create wells. Allow the gel to solidify.
  d. Remove the comb and place the gel in an electrophoresis chamber filled with 1x TAE buffer.
  e. Load PCR products from both PCR#1 and PCR#2 into separate wells.
    ***Note:*** Load a 1 kb DNA ladder into one well as a size reference.
  f. Run the gel at 100 V for approximately 45 min or until clear separation of DNA bands is visible.
  g. Visualize the DNA bands under UV light using a gel documentation system.
  h. Identify the ∼500 bp bands corresponding to the expected PCR products. Carefully excise the ∼500 bp DNA bands using a clean scalpel.
  i. Extract DNA from the gel slices using the Monarch DNA Gel Extraction Kit (NEB, Cat# T1020S), following the manufacturer’s instructions (https://www.neb.com/en-us/protocols/2024/07/11/standard-gel-extraction-protocol-using-the-monarch-spin-dna-gel-extraction-kit-and-centrifugation).
  j. Submit the purified DNA samples for sequencing to a provider such as MCLAB.

## Expected outcomes

**Figure 7:**
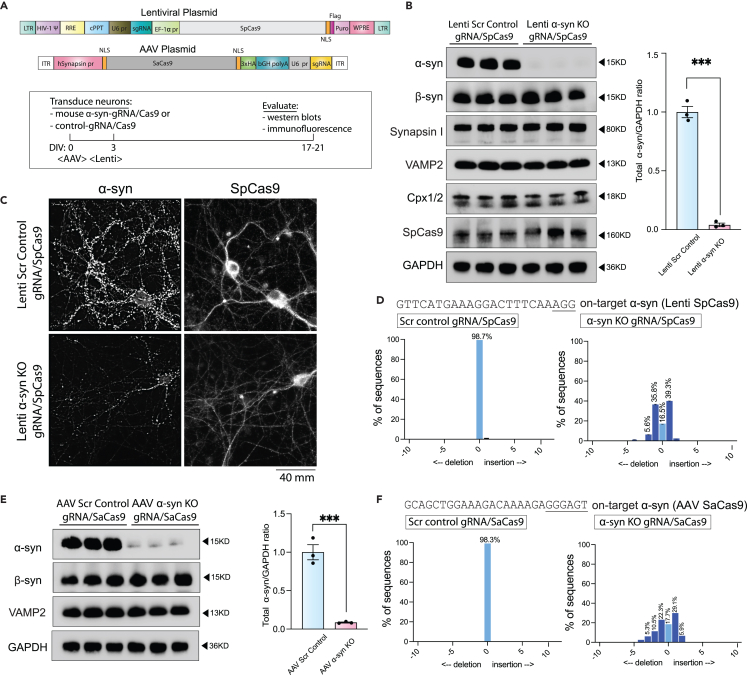
Global and selective attenuation of α-syn in hippocampal cultures using CRISPR-Cas9 (A) Cultured neurons were transduced with lentiviruses (13,013 bp) or AAVs (7289 bp) carrying CRISPR-components (α-syn KO gRNA and Sp/SaCas9) to knockdown mouse α-syn, followed by western blots and immunostaining. A gRNA not targeting to any known sequence in the mouse genome [“Scramble (Scr) Control”] was used as controls. (B) Endogenous mouse α-syn was almost undetectable in lentiviral-transduced cultures using western blots, and the attenuation was selective for α-syn (gel and quantification on the right; ∗∗∗, *p* < 0.001 by Student’s *t* test; n = 3). (C) Representative images show marked attenuation of synaptic α-syn immunostaining in lentiviral-transduced α-syn KO-gRNA/SpCas9 treated neurons, with widespread (∼100%) transduction of SpCas9. (D) On target TIDE analysis of DNA from mouse hippocampal neurons treated with lentiviral-transduced α-syn KO/Cas9-gRNA (n = 2 samples). Note on-target deletions and insertions seen only with α-syn-gRNA/SpCas9. (E) Western blots from cultured neurons transduced with a single AAV carrying α-syn KO-gRNA and SaCas9 show marked attenuation of α-syn levels, quantified on the right (∗∗∗, p < 0.001 by Student’s t-test; n = 3).

A key goal of this protocol is to globally attenuate α*-*syn in virtually all neurons within a given coverslip. This not only ensures uniform attenuation of the gene of interest, but is also experimentally desirable as other constructs can be transfected or transduced in the KO background (for example probes to evaluate synaptic function^1^). Successful CRISPR KO of α*-*syn by both lentiviral and AAV-based approaches should lead to a substantial reduction in α*-*syn protein levels, as demonstrated in Figure 7 using western blotting and immunostaining. Vector maps and general strategies for transduction are outlined in Figure 7A. Specificity of knockdown is another key goal. Expression levels of other synucleins should remain unchanged (note that γ*-*synuclein is not detectable in hippocampal neurons^1^). Moreover, there should be no compensatory changes in key α-syn binding partners, such as VAMP2 and synapsin, or other synaptic proteins known to be dysregulated in KO mice lacking all synucleins (Figure 7B). Along with α*-*syn knockdown, transduction efficiency should also be assessed in every experiment by immunostaining for Cas9 (Figure 7C). The α-syn-specific sgRNA sequence 5′-GGTTCATGAAAGGACTTTCAA-3’ (Lenti SpCas9 α-syn KO) achieved editing efficiencies ranging from 61-94% (Figure 7D). Marked knockdown of α-syn was also seen with AAV SaCas9 α-syn KO (Figure 7E), with high levels of on-target editing (Figure 7F). Minimal off-target effects were seen by TIDE analysis (Figure S1).

**Figure 8:**
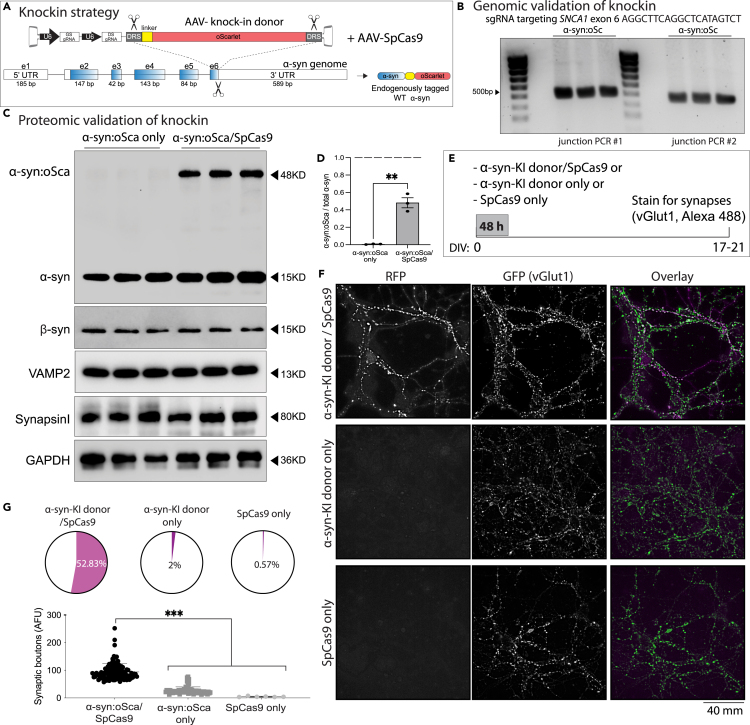
Endogenous tagging of α-syn in cultured neurons using CRISPR-Cas9 (A) KI strategy to insert a fluorescent tag oScarlet at the C-terminus of endogenous mouse α-syn. Note that two AAVs are used – the AAV α-syn:oScarlet KI donor and AAV-SpCas9. CRISPR KI targeting of the *Snca*1 exon 6 locus was achieved using a sgRNA sequence (5′-AGGCTTCAGGCTCATAGTCT-3′) cloned into an AAV KI donor vector containing the oScarlet tag. Hippocampal neurons were transduced with the AAV, and genomic DNA was extracted at DIV 21. (B) PCR analysis was performed to validate the integration at the 5′ and 3′ junctions. For the 5′ junction, amplification was conducted using the α-syn forward primer (PCR#1): 5′ TGTGCTTTCTCTTCCCTCTCTG 3′ and oScarlet reverse primer: 5′ ACAGGATGTCCCAGGAGAAG 3’. For the 3′ junction, the α-syn forward primer (PCR#2): 5′ ATAACACTTCGTGCAGCACC 3′ and the same oScarlet reverse primer were used. Amplified products of approximately 500 bp confirm successful KI integration at both junctions. (C) Samples from CRISPR KI tagging (neuronal protein lysates) were analyzed by NuPAGE and immunoblotted with antibodies against α-syn, β-syn (CRISPR KI specificity control), VAMP2/Synapsin I (α-syn interacting proteins) and GAPDH as loading control. Note higher molecular-weight band corresponding to oScarlet-tagged α-syn. (D) Western blotting quantification for gels in (C); ∗∗, *p* < 0.001 by Student’s *t* test (n = 3). (E) Strategy for evaluating oScarlet-KI. The AAV α-syn:oScarlet KI donor only, or AAV SpCas9 only were used as controls. AAV transduced neurons were stained with vGlut1 (green) to identify synapses. (F) Representative images of cultured neurons expressing the KI construct (α-syn-KI donor/SpCas9), or controls. Note that neurons co-transduced with AAV α-syn:oScarlet KI donor/AAV SpCas9 show a punctate staining pattern that overlaps with a subset of synapses (top panels). However, no synaptic staining was seen with either the AAV α-syn:oScarlet KI donor only, or AAV SpCas9 alone. (G) Comparison of synaptic fluorescence intensity across all conditions. Notably, α-syn was endogenously tagged with oScarlet in approximately 53% of synapses (top). Minimal synaptic fluorescence was observed in control conditions using either the AAV α-syn:oScarlet KI donor alone or AAV SpCas9 alone (∗∗∗, p < 0.001 by Student’s t-test; AAV α-syn:oScarlet KI donor + AAV SpCas9: n = 106 synapses; AAV α-syn:oScarlet KI donor only: n = 68; AAV SpCas9 only: n = 6).

In CRISPR-KI experiments, a vector carrying the AAV α-syn:oScarlet KI donor template is designed to integrate the oScarlet tag at the C-terminus of α-syn in cultured hippocampal neurons (when AAV SpCas9 is also co-transduced, see Figure 8A for strategy). PCR amplification of the 5′ and 3′ integration junctions, followed by Sanger sequencing, should confirm integration of the tag (Figure 8B and Parra-Rivas et al. 2023^1^). Western blot analysis of lysates from neurons treated with the α-syn CRISPR KI tools should reveal two bands: a higher band (∼48 kDa) corresponding to α-syn tagged with oScarlet, and a lower band (∼15 kDa) representing unmodified α-syn in neurons that did not undergo KI (Figure 8C, top gel). Note that protein levels of β-synuclein, VAMP2, Synapsin I, and GAPDH should remain consistent across all conditions (Figure 8C). Synaptic localization of endogenous α*-*syn tagged with oScarlet can be visualized by routine fluorescence microscopy (Figures 8D–8F). Note that in our hands, ∼53% of glutamatergic synapses (marked by VGLUT1) show oScarlet expression (Figure 8G). Previous studies suggest that donor-templates in such experiments show leaky expression,^24^ perhaps due to promoter-like activity of AAV-ITR sequences.^25^^,^^26^ However, transduction with only the AAV α-syn:oScarlet KI donor (with o-Scarlet) showed minimal synaptic fluorescence (Figure 8G), though we did see very low levels of fluorescence in a few cell bodies and neurites, suggesting some leaky expression (Figure S2).

## Limitations

Viral transduction can trigger immune responses or stress pathways in cells that can lead to toxicity, decreased neuronal viability, and other phenotypes unrelated to gene editing. Careful adherence to the protocol and evaluation of neuronal health should alleviate these concerns. On the other hand, knockdown of an essential gene can cause morphologic changes or decreased cell viability (on-target effect), and initial pilot studies should carefully evaluate development and synaptic maturation in the absence of the gene of interest. For knockdown of genes that have related homologues or isoforms, putative compensation should be evaluated. Additionally, protein levels of at least a few interactors should also be evaluated to rule out other compensatory changes. Off-target effects of CRISPR-Cas9 can be alleviated by designing specific gRNAs and in-silico/TIDE analyses as described in the protocol. Though these methods are sufficient for routine functional assays, further in-depth genome-scale off-target analysis (typically relevant for preclinical studies) can also be done using techniques such as GUIDE-Seq or CHANGE-Seq.^27^^,^^28^

We also used AAV-based CRISPR-KI (HiUGE) to insert the o-Scarlet tag at the C-terminal end (last exon) of α-syn, using an intracellularly linearized single homology arm donor. This intron-targeting strategy minimizes disruption of essential coding sequences; however, it may reduce KI efficiency compared to exon-targeting approaches, as exons are typically more transcriptionally active and accessible to the repair machinery. Another issue with this approach is the leaky expression of the o-Scarlet tag in the KI donor, as discussed previously. However, though there was only mild leaky expression of the donor-tag in neuronal cell-bodies, with no mis-expression in synapses (Figures 8D–8G; S2). Finally, although HiUGE is designed to minimize off-target effects, the potential for unwanted DNA cleavage or integration at unintended sites persists. These off-target effects can lead to genomic instability or affect other genes, complicating interpretation. At minimum, we recommend genomic and proteomic validation to ensure KI at the correct locale, as well as visual confirmation of synaptic targeting, as shown in Figure 8. Additionally, it is important to generate multiple KI donors using different sgRNA sequences and compare localization and synaptic targeting. Discrepancies in localization across sgRNAs may necessitate designing and testing additional sgRNAs targeting either the C-terminus or N-terminus to identify the best KI strategy.

## Troubleshooting

### Problem 1

Low genome editing efficiency due to suboptimal gRNA design.

### Potential solution

• Use validated algorithms for gRNA design. To maximize editing efficiency, we recommend using well-established CRISPR design tools such as CRISPick (as described in this protocol), or alternatively platforms like CRISPOR, Benchling, or CHOPCHOP. These tools evaluate multiple parameters, including on-target activity scores, predicted off-target effects, and other sequence features that influence guide performance.
• Prioritize gRNAs with high on-target activity scores. Select gRNAs with high predicted cleavage activity and minimal predicted off-target effects. Favor guides that have strong predicted efficacy and at least three mismatches relative to any off-target sites in coding regions.
• Target early exons: For gene KOs, designing gRNAs targeting early coding exons increases the chance of generating frameshift mutations that result in complete loss of function. If multiple isoforms exist, target exons common to all major transcripts.
• Avoid guides with extreme GC content. Optimal gRNAs typically have a GC content between 40–60%. Very low or very high GC content can negatively affect Cas9 binding and cleavage efficiency.
• Test multiple candidates. We strongly recommend designing and testing at least 2–5 independent gRNAs per target site. This increases the likelihood of identifying a guide with high editing efficiency.

### Problem 2

• Viral Production Issues.
• Successful delivery of CRISPR-Cas9 components to post-mitotic neurons relies heavily on efficient viral packaging, as traditional transfection methods are not suitable for these cells. In case there are issues in generating high-titer lentivirus or AAV, consider the following troubleshooting strategies:

### Potential solution

• Verify Plasmid Integrity. Ensure all plasmid constructs used for packaging (e.g., transfer vector, helper plasmids, and envelope or capsid plasmids) are correctly assembled and free of mutations. Sequence verification and restriction enzyme digestion can confirm integrity before transfection.
• Optimize Transfection Conditions. The efficiency of viral production is highly dependent on the health and confluency of HEK293T cells at the time of transfection. Cells should be 70–80% confluent, healthy, and not over-passaged. Use a transfection reagent with high efficiency in HEK cells, such as PEI Max or Lipofectamine 3000, and optimize DNA:reagent ratios. Use endotoxin-free plasmid DNA, and ensure that your transfection mixes are incubated appropriately before adding to cells.
• Monitor Cell Health and Morphology. Post-transfection, HEK293T cells should appear healthy and exhibit signs of viral production (e.g., slight cell rounding or detachment over time). Poor cell health can lead to drastically reduced viral yields. Replace the culture medium 12–16 h after transfection to minimize cytotoxicity.
• Improve Viral Harvesting. For lentivirus, collect supernatant at 48 and 72 h post-transfection. These time points generally yield the highest concentration of viral particles while maintaining particle integrity. Extending the collection time is not recommended, as prolonged incubation can lead to increased media acidification, which can negatively impact viral infectivity and stability. For AAV, harvest the cells approximately 72 h post-transfection, as viral yield typically peaks around this time in HEK293T cells. Delaying harvest beyond 72 h can result in increased cell death, enzymatic degradation, and reduced viral integrity, ultimately lowering AAV yield and infectivity.
• Use Alternative Serotypes or Promoters. The choice of AAV serotype can significantly impact transduction efficiency, especially in neurons. For example, AAV9 often yields better neuronal expression than AAV2, but in our hands, PHP.eB consistently achieves nearly 100% transduction efficiency in cultured neurons. PHP.eB has also been shown to outperform AAV9 in targeting the central nervous system of non-human primates (NHPs).^29^ In addition, the promoter used to drive transgene expression plays a critical role in specificity and expression levels. For neuronal applications, promoters such as human Synapsin offer greater specificity and sustained expression compared to ubiquitous promoters like CMV. Promoter selection should be optimized based on the target cell type and experimental goals.

### Problem 3

Poor CRISPR KO in neurons.

### Potential solution

• Optimize Viral Titer. Ensure that the viral titer is high enough to achieve efficient transduction in a sufficient number of neurons. In our experiments, we consistently observed ∼100% transduction efficiency and successful gene editing in cultured neurons using a MOI of 5 for lentivirus (Parra-Rivas et al., 2023^1^) and MOI of 100,000 for AAVs. Importantly, we found that viral doses could be safely increased to MOI 8 (lentivirus) and MOI 200,000 (AAV) without signs of toxicity. However, optimal MOI may vary depending on the experimental context, so titration experiments are recommended to balance transduction efficiency with cell health.
• Adjust Incubation Time. Extending the incubation period can enhance viral transduction and CRISPR editing efficiency. For lentiviral transductions, we recommend limiting incubation to no more than 7 h, as prolonged exposure may compromise neuronal viability. For AAV transductions, a 48-h incubation period is typically optimal—extended exposure beyond this point can increase cytotoxicity. Regardless of the viral system, careful monitoring of cell health throughout the incubation period is essential to avoid unintended toxicity.
• Transduction Timing and Neuronal Age. The age of neurons at the time of transduction is a key factor in achieving optimal CRISPR editing. For lentiviral transductions, we observed the highest KO efficiency when transductions were performed at DIV 3. Transductions at DIV 5 or later resulted in a marked decline in editing success, likely due to reduced viral susceptibility in more mature neurons. For AAV transductions, we recommend infecting neurons 2–5 h after plating for optimal uptake. However, if early-stage cultures show sensitivity to AAV exposure, delaying transduction to DIV 1–3 can still yield robust results with reduced toxicity.

### Problem 4

Low neuronal viability after lentiviral transduction.

### Potential solution

Many commercially available reagents used to concentrate or purify lentiviral particles can be toxic to neurons, and thorough washing of cultures after transduction is important to minimize this toxicity. A recommended approach is to add 1 mL of fresh NB/B27 medium to the culture and then carefully remove 1 mL of the media (4 times). Repeating this washing step 6 to 8 times will help ensure the complete removal of any residual lentiviral particles or toxic reagents that could compromise neuronal viability. Additionally, increasing the total volume of media during transduction can help dilute viral particles and any residual toxins, reducing stress on the neurons. While we typically use 1 mL of media during transduction, increasing this to 1.5 mL can mitigate toxicity and improve neuronal survival when cell death is observed. If further reduction in toxicity is needed, lowering the titer to MOI = 3 can also help. However, this may reduce the transduction efficiency, so a balance between titer and cell health needs to be considered.

### Problem 5

Toxicity in neurons after AAV transduction.

### Potential solution

Several factors can help minimize AAV-induced toxicity in neurons such as lowering the titer, increasing media volume to dilute viral particles, reducing duration of exposure, and increased washing post-transduction.

### Problem 6

Non-specific expression of AAV α-syn:oScarlet KI donor (leaky expression).

### Potential solution

In the absence of AAV spCas9, AAV α-syn:oScarlet KI donor should not integrate into the genome. Consistent with this expectation, we only observed minimal leaky expression in a subset of somata and neurites. If non-specific expression is noticeable, we recommend reducing the AAV titer by half and shortening the transduction duration, both of which can help limit non-specific uptake and expression. Importantly, the low-level expression we observed was confined to a few cells and was not detectable by Western blot (Figure 8C), indicating that leaky expression is typically minor and unlikely to affect downstream analyses.

## Resource availability

### Lead contact

Further information and requests for resources and reagents should be directed to the lead contact, Subhojit Roy (sroy@ucsd.edu).

### Technical contact

Technical questions on executing this protocol should be directed to the technical contact, Leonardo Parra-Rivas (l2parra@health.ucsd.edu).

### Materials availability

All reagents generated in this study are available from the lead contact with a completed material transfer agreement.

### Data and code availability

Original data has been deposited in Zenodo (DOI: https://doi.org/10.5281/zenodo.14405588).

## Acknowledgments

This work was supported by grants to S.R. from the NINDS (R01NS111978) and the Farmer Family Foundation. This research was also funded in whole or in part by Aligning Science Across Parkinson’s (ASAP-020495) through the Michael J. Fox Foundation for Parkinson’s Research (MJFF). For the purpose of open access, the author has applied a CC BY public copyright license to all Author Accepted Manuscripts arising from this submission. L.A.P.-R. was supported by a postdoctoral fellowship from the American Parkinson’s Disease Association (APDA) and the Parkinson’s Foundation Launch award PF-LAUNCH-1046253.

## Author contributions

L.A.P.-R. and S.R. conceived the study. L.A.P.-R. wrote the original draft and created the figures. L.A.P.-R. and S.R. jointly prepared the final manuscript. L.A.P.-R. and R.S. conducted the biochemical and immunohistochemistry experiments and analyses. Off-target studies were performed and analyzed by T.E.R., H.O.B., and J.C.-S. under the supervision of M.J.Z. Y.O. developed the CRISPR knockin tools and handled AAV production. All authors reviewed and approved the final manuscript.

## Declaration of interests

The authors declare no competing interests.
