# Supplementary material for: Protocol for CRISPR-based manipulation and visualization of endogenous α-synuclein in cultured mouse hippocampal neurons
Source: STAR Protoc. 2025 Jul 21;6(3):103945. doi: 10.1016/j.xpro.2025.103945 (PMC12305205; doi:10.1016/j.xpro.2025.103945)
Supplement: Document S1. Figures S1 and S2 and Table S1 [file mmc1.pdf]

SUPPLEMENTAL INFORMATION

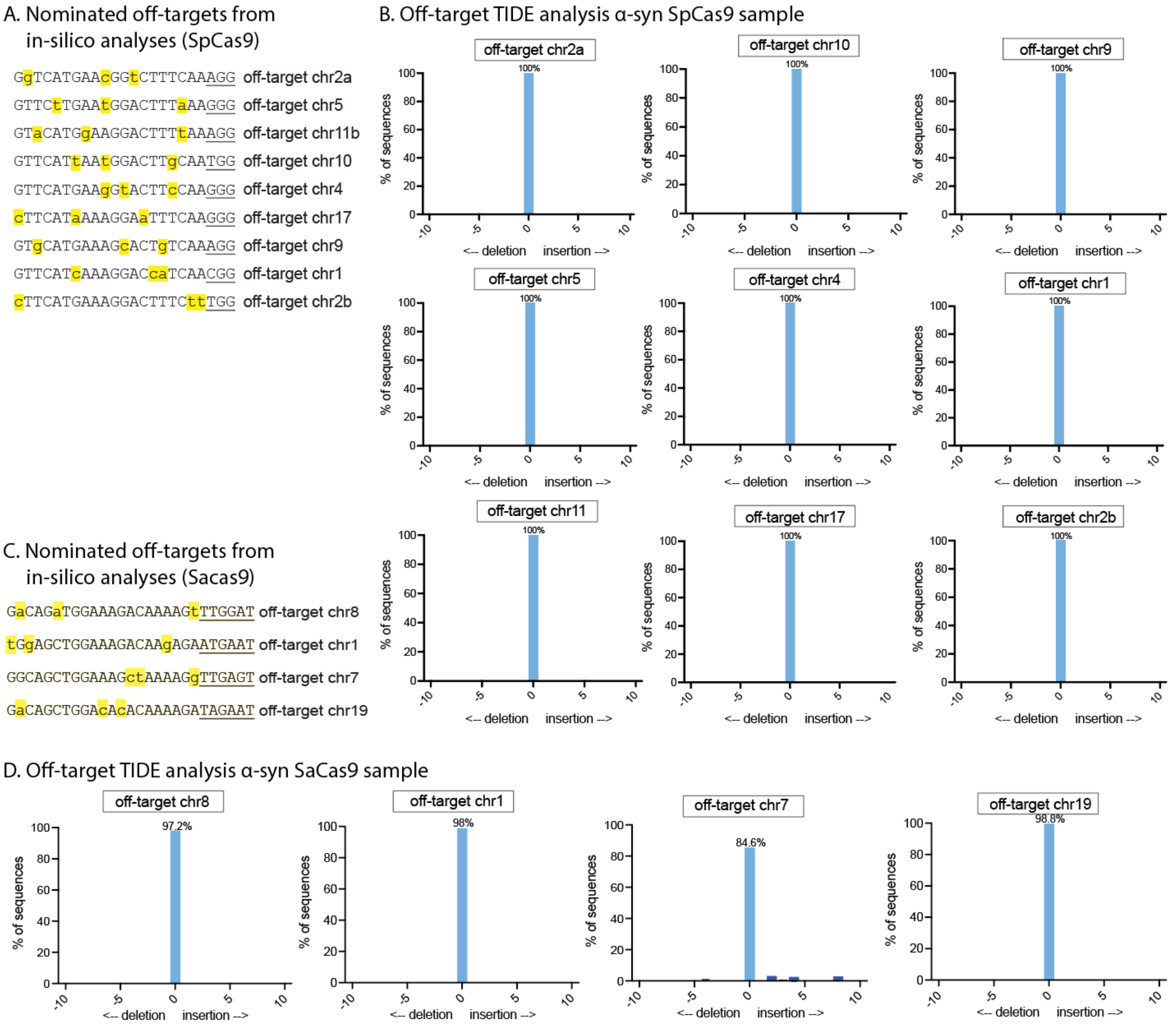

<u>Supplementary Fig.1</u>: TIDE analysis of *in-silico* nominated off-target editing, related to Step 8

(A) Nominated α-syn-gRNA/SpCas9 off-target sites from *in-silico* analyses containing $\leq$ 3 mismatches. Nucleotide mismatches are lowercase and highlighted in yellow, and the PAM sites are underlined.

(B) Off-target TIDE analysis for the nominated sites was performed on samples from neurons treated with lentiviral-transduced α-syn-gRNA/SpCas9 (n = 2). Note that no indels were seen for any of the nominated sites.

(C) Nominated α-syn-gRNA/SaCas9 off-target sites from *in-silico* analyses containing $\leq$ 3 mismatches. Nucleotide mismatches are lowercase and highlighted in yellow, and the PAM sites are underlined.

(D) Off-target TIDE analysis for the nominated sites was performed on samples from neurons treated with AAV-transduced α-syn-gRNA/SaCas9 (n = 1). Note that only minimal indels were seen for one of the nominated sites (chr7).

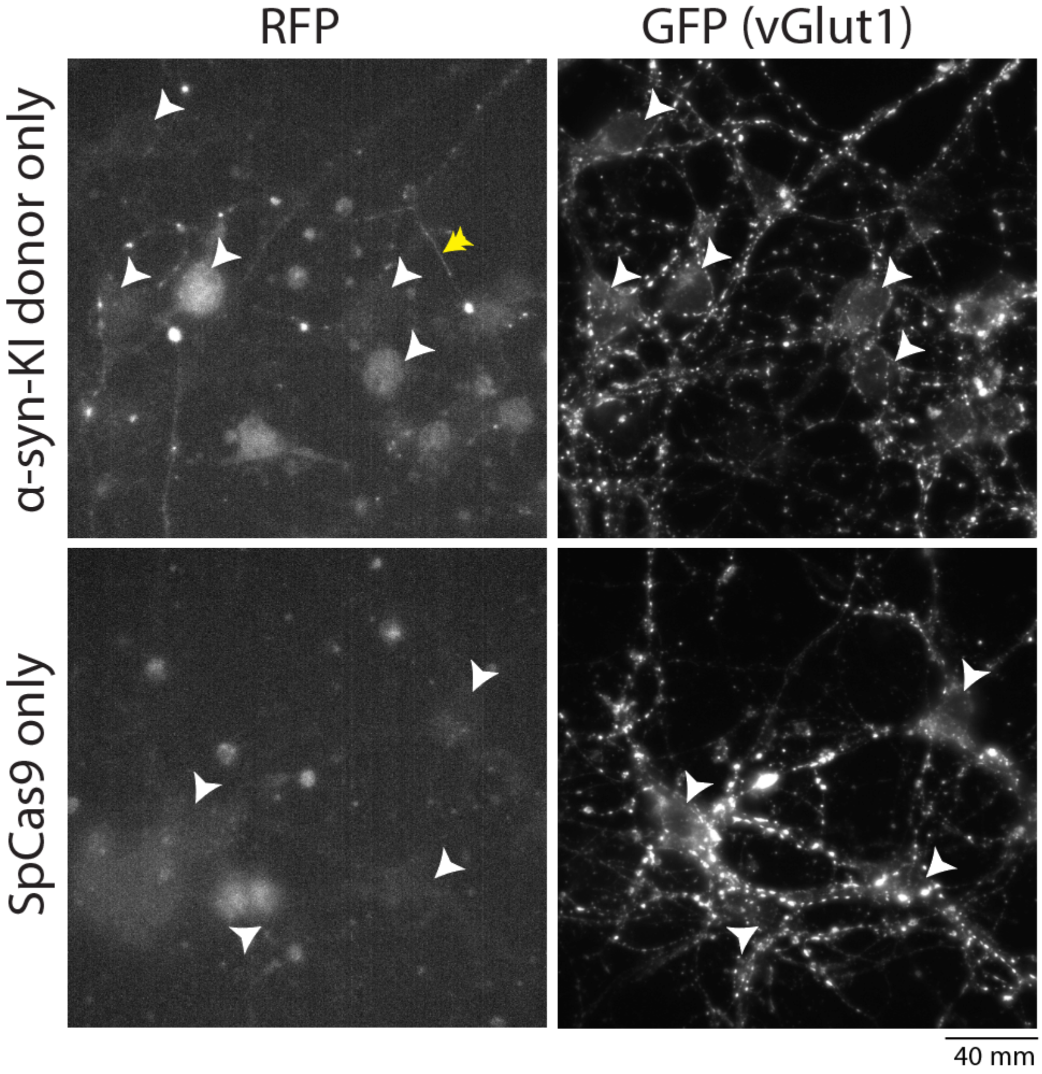

<u>Supplementary Fig. 2</u>: Minimal leaky expression of the α-syn donor construct, related to Step 7

Cultured neurons were transduced with AAVs carrying either the AAV α-syn:oScarlet KI (α-syn-KI) donor construct (with o-Scarlet), or AAV-SpCas9 (SpCas9). Note that in principle, o-Scarlet fluorescence should only be seen when both AAVs are co-transduced, but we saw faint o-Scarlet fluorescence in some neuronal cell bodies and neurites expressing only the AAV α-syn:oScarlet KI donor construct, suggesting low levels of leaky expression (single-arrowheads – soma, double-arrowheads – neurite; upper panels). No fluorescence was seen with AAV SpCas9 only, as expected (lower panels, single-arrowheads mark neuronal soma). Note that despite this low-level of fluorescence in some cell-bodies and neurites, no synaptic expression is seen with the α-syn-KI donor construct (see **Fig. 8F** – middle panels).

PLASMID NAME	SOURCE	IDENTIFIER	PURPOSE
AAV SaCas9	Addgene	RRID:Addgene_61591	Backbone AAV plasmid for SaCas9-based CRISPR KO of mouse α-syn and Scramble Control
psPAX2	Addgene	RRID:Addgene_12260	Lentiviral packaging plasmid
pMD2.G	Addgene	RRID:Addgene_12259	Lentiviral packaging plasmid
LentiCRISPR v2	Addgene	RRID:Addgene_52961	Backbone Lentiviral plasmid for SpCas9-based CRISPR KO of mouse α-syn and Scramble Control
PX552	Addgene	RRID:Addgene_ 60958	Backbone AAV vector for SpCas9-mediated CRISPR KI of oScarlet at the C-terminus of mouse α-syn.
AAV SpCas9	Addgene	RRID:Addgene_60957	AAV vector for SpCas9-mediated CRISPR KI
pPHP.eB	Addgene	RRID:Addgene_103005	AAV packaging plasmid
pHelper	Agilent Technologies	Cat # 240071	AAV packaging plasmid
pMJ114	Addgene	RRID:Addgene_85995	Backbone for sgRNA scaffold PCR based amplification
pMJ117	Addgene	RRID:Addgene_85997	Backbone for human U6 promoter PCR based amplification
pMJ179	Addgene	RRID:Addgene_85996	Backbone for mouse U6 promoter PCR based amplification
AAV α-syn:oScarlet KI donor (pLP857)	This study	RRID:Addgene_ 239403	AAV vector for SpCas9-mediated CRISPR KI of oScarlet at the C-terminus of mouse α-syn
Lenti SpCas9 Scramble Control (pLP16)	This study	RRID:Addgene_ 239417	Negative control Lentiviral plasmid for SpCas9-based CRISPR KO
Lenti SpCas9 α-syn KO (pLP17)	This study	RRID:Addgene_ 239418	Lentiviral plasmid for SpCas9-based CRISPR KO of mouse α-syn
AAV SaCas9 Scramble Control (pLP110)	This study	RRID:Addgene_ 239419	Negative control AAV vector for SaCas9-based CRISPR KO
AAV SaCas9 α-syn KO (pLP111)	This study	RRID:Addgene_ 239420	AAV vector for SaCas9-based CRISPR KO of mouse α-syn

<u>Supplementary table.1</u>: Table summarizing the different plasmids used in this study, related to Step 1.
